# A multi-functional PEGylated gold(iii) compound: potent anti-cancer properties and self-assembly into nanostructures for drug co-delivery†
†Electronic supplementary information (ESI) available: Experimental details, ^1^H NMR and MALDI-TOF-MS of **1** and **2**; TEM image, DLS profile and zeta potential profile of **2**; zeta potential profiles of **1** and **NC1**; cell viability profiles after treatment of the gold(iii) complexes and nanocomposites; total-ion chromatograms of UPLC-QTOF-MS of **1**, **3** and **4**; cellular uptake of the gold(iii) complexes; fluorescence microscopy images and flow cytometric analysis of the assay with FITC-Annexin V and propidium iodide; time-dependent fluorescence microscopy images and flow cytometric analysis of the assay using CellEvent™ Caspase-3/7 Green ReadyProbes Reagent; fluorescence microscopy images and flow cytometric analysis of the co-culture model of HCT116 and NCM460 cells; selected-ion chromatograms from UPLC-QTOF-MS of homogenized tumor tissues of mice treated by **1**; biodistribution of gold complexes in nude mice bearing HCT116 xenografts; UPLC traces of the nanocomposites; tables showing the relative toxicities of the gold(iii) complexes and nanocomposites toward cancer cells over non-tumorigenic cells. See DOI: 10.1039/c6sc03210a



**DOI:** 10.1039/c6sc03210a

**Published:** 2016-11-22

**Authors:** Clive Yik-Sham Chung, Sin-Ki Fung, Ka-Chung Tong, Pui-Ki Wan, Chun-Nam Lok, Yanyu Huang, Tianfeng Chen, Chi-Ming Che

**Affiliations:** a State Key Laboratory of Synthetic Chemistry , Department of Chemistry and Chemical Biology Centre , The University of Hong Kong , Pokfulam Road , Hong Kong , China . Email: cmche@hku.hk; b Department of Chemistry , Jinan University , Guangzhou 510632 , China

## Abstract

Gold(iii) porphyrin–PEG conjugates [Au(TPP–COO–PEG_5000_–OCH_3_)]Cl (**1**) and [Au(TPP–CONH–PEG_5000_–OCH_3_)]Cl (**2**) have been synthesized and characterized. Based on the amphiphilic character of the conjugates, they were found to undergo self-assembly into nanostructures with size 120–200 nm and this did not require the presence of other surfactants or components for nano-assembly, unlike most conventional drug nano-formulations. With a readily hydrolyzable ester linkage, chemotherapeutic [Au(TPP–COOH)]^+^ exhibited triggered release from the conjugate **1** in acidic buffer solution as well as *in vitro* and *in vivo* without the formation of toxic side products. The nanostructures of **1** showed higher cellular uptake into cancer cells compared to non-tumorigenic cells, owing to their energy-dependent uptake mechanism. This, together with a generally higher metabolic rate and more acidic nature of cancer cells which can lead to faster hydrolysis of the ester bond, afforded **1** with excellent selectivity in killing cancer cells compared with non-tumorigenic cells *in vitro*. This was corroborated by fluorescence microscopy imaging and flow cytometric analysis of co-culture model of colon cancer (HCT116) and normal colon (NCM460) cells. *In vivo* experiments showed that treatment of nude mice bearing HCT116 xenografts with **1** resulted in significant inhibition of tumor growth and, more importantly, minimal systemic toxicity as revealed by histopathological analysis of tissue sections and blood biochemisty. The latter is explained by a lower accumulation of **1** in organs of treated mice at its effective dosage, as compared to that of other gold(iii) porphyrin complexes. Co-assembly of **1** and doxorubicin resulted in encapsulation of doxorubicin by the nanostructures of **1**. The nanocomposites demonstrated a strong synergism on killing cancer cells and could overcome efflux pump-mediated drug-resistance in a doxorubicin-resistant ovarian cancer cell line (A2780adr) which was found in cells incubated with doxorubicin alone. Also, the nanocomposites accumulated more slowly in non-tumorigenic cells, resulting in a lower toxicity toward non-tumorigenic cells. These results indicate the potential application of **1** not only as an anti-cancer agent but also as a nanoscale drug carrier for chemotherapy.

## Abstract 2

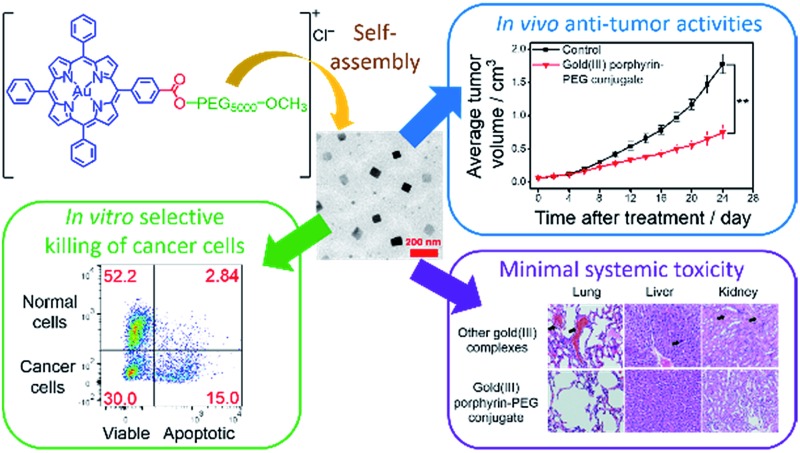
Amphiphilic gold(iii) porphyrin–PEG conjugates can undergo self-assembly into nanostructures, resulting in promising anti-cancer properties with minimal systemic toxicity. The nanostructures can be employed as nanocarriers for drug co-delivery.

## Introduction

Gold compounds have received growing interest as chemotherapeutics in the past few decades.^1–7^ Unlike cisplatin and many other platinum-based drugs which exhibit anti-cancer properties primarily by targeting DNA,^3,8,9^ gold(i) and/or gold(iii) complexes have demonstrated a variety of actions, including inhibition of thioredoxin reductase, direct DNA damage, alteration of cell cycles, proteasome inhibition and modulation of specific kinases.^1,3–7,10–20^ These multifaceted modes of action are important for gold complexes to display potent cytotoxicity against cancer cells, particularly toward cisplatin- and multidrug-resistant cell lines.^3–7,15^ Among these gold compounds, gold(iii) porphyrin complexes [Au(TPP)]Cl (H_2_TPP = 5,10,15,20-tetraphenylporphyrin and its derivatives) show excellent stability under physiological conditions and only weak interactions with serum proteins,^6,7,13^ in contrast to other gold complexes which decompose quite readily in biological media.^2^ Stability is believed to be crucial for the promising *in vivo* anti-cancer activities of gold(iii) porphyrin complexes against different types of carcinoma.^6,7,14,15^ As is commonly encountered with metallodrugs,^3,21^ the main hurdles in the development of clinically used gold(iii) porphyrin complexes for chemotherapy are their poor bioavailability and high toxicity in normal cells and tissues.^6,7^

Nano-formulation^22–32^ can be a potential solution to the challenges found in the *in vivo* application of anti-cancer metal complexes.^22–28^ Nanostructures of around 100 nm have been found to accumulate in the tumor vicinity due to the enhanced permeability and retention (EPR) effect,^22–32^ which originates from the leaky tumor vasculatures and impaired lymphatic system in solid tumors. Therefore, a significant improvement in biodistribution of the drug molecules and a concomitant reduction in toxic side effects can be accomplished by the nanostructures when compared to traditional therapeutics.^22,23,29–32^ One of the conventional approaches for nano-formulation is to encapsulate drug molecules in nanoscale drug carriers. For example, cisplatin and its platinum(iv) prodrugs have been encapsulated by biodegradable polymeric micelles, liposomes and nanogels.^22–24,26–28^ Nano-formulations from the conjugation of platinum complexes onto preformed nanostructures, such as gold nanoparticles^25^ and magnetic iron oxide nanoparticles,^28^ have also been described. Both methodologies have been demonstrated to develop nanostructures with superior *in vitro* and/or *in vivo* anti-cancer efficacy. However, in view of the skin toxicity that arose from the nanocarrier of Doxil (liposomal formulation of doxorubicin),^33^ the toxicity of the components utilized for nano-formulation, including those which have been approved by the US Food and Drug Administration (FDA) for drug formulation, has received growing concern.^29,31,34^ As a result, the development of nanostructures of metallodrugs with minimal content of the components for nano-assembly would be an attractive strategy for chemotherapy.

Metal complexes with sterically unhindered structures can undergo self-assembly into nano- or microstructures without the aid of nanocarriers or preformed nanostructures.^18,35–46^ This property can be explained by the amphiphilic character of the metal complexes that gives rise to strong non-covalent interactions for the formation of sophisticated nano- or microstructures.^18,35–46^ As a number of cytotoxic metal complexes contain hydrophobic π-conjugated ligands,^6,7,11–21,36,43^ it is anticipated that an introduction of hydrophilic components onto these metal complexes could render them with amphiphilic properties, and hence will increase their solubility in aqueous solutions and facilitate the formation of nanostructures for *in vivo* accumulation in tumors by the EPR effect. Poly(ethylene glycol) (PEG), which is a hydrophilic polymer approved by the FDA for drug formulation,^47^ is an excellent candidate for the development of amphiphilic cytotoxic metal complexes. This is not only because of its hydrophilic character leading to PEGylated metal complexes having a good aqueous solubility, but also due to its known antifouling properties, *i.e.* to provide steric bulk which minimizes the interactions of the metal complexes with proteins or other biomolecules in blood serum in order to prevent rapid clearance of the complexes, which is commonly found in drug molecules without PEG.^47^ Although PEG–drug conjugation is a common methodology in drug administration for improving efficacy, the formation of nanostructures from PEG–drug conjugates without the aid of nanocarriers or other components for nano-assembly has rarely been reported.^48^ In view of the strong tendency of amphiphilic metal complexes to form highly-ordered structures, we were prompted to design and synthesize gold(iii) porphyrin–PEG conjugates. Through judicious choice of porphyrin ligands and linkers for PEG conjugation, the gold(iii) porphyrin–PEG conjugates could form interesting nanostructures in aqueous media, and the anti-cancer gold(iii) porphyrin moiety can be released from the conjugates in cancer cells for effective killing of the cancer cells without the formation of toxic side products. Moreover, the nanostructures of gold(iii) porphyrin–PEG conjugates could be further utilized as nanoscale drug carriers for other chemotherapeutics to achieve a strong synergistic effect on killing cancer cells by the co-delivery, as well as minimizing toxic side effects and overcoming drug resistance found in the administration of the chemotherapeutic alone. Herein is described the self-assembly and anti-cancer properties of multifunctional gold(iii) porphyrin–PEG conjugates.

## Results and discussion

### Design, synthesis and characterization

**Scheme 1:**
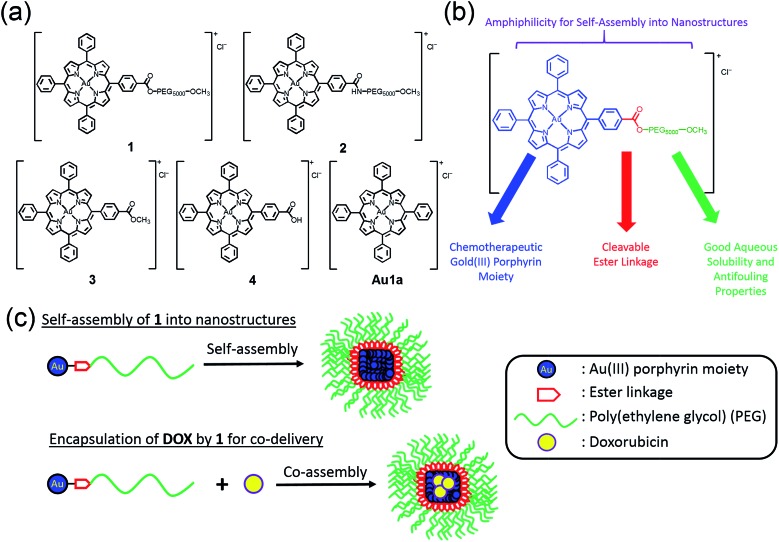
(a) Chemical structures of **1–4** and **Au1a**. (b) Multi-functional properties of **1**. (c) Schematic cartoon showing the preparations of nanostructures of **1** and nanocomposites of **1** and **DOX** by the self-assembly of **1**.

The chemical structures of complexes **1–4** and **Au1a** are depicted in Scheme 1. The gold(iii) porphyrin–PEG conjugates are decorated with multiple functionalities for their anti-cancer properties. The PEG pendant can render gold(iii) porphyrin complexes with good aqueous solubility while lessening the reactivity of the complex with biomolecules. Importantly, PEG also provides complexes with amphiphilic character so that the gold(iii) porphyrin–PEG conjugates can undergo self-assembly into nanostructures in aqueous media. These properties are important for improving the *in vivo* efficacy of gold(iii) porphyrin complexes for anti-cancer treatment with reduced toxic side effects. To avoid significantly lowering the cytotoxicity of the gold(iii) porphyrin complexes by the PEG pendant as observed in other PEG–drug conjugates,^47^ the ester linkage, which can be hydrolyzed readily by intracellular esterases and/or acidic conditions in the tumor vicinity,^34,49^ was employed to conjugate the PEG and gold(iii) porphyrin moiety (complex **1**). The amide counterpart, **2**, was also synthesized and examined to understand the contribution of the cleavable ester linkage on the anti-cancer properties of gold(iii) porphyrin–PEG conjugates. It is noteworthy that hydrolysis of the conjugates would only release the anti-cancer gold(iii) porphyrin moiety and the hydrolyzed product of FDA approved PEG. Therefore, no additional toxicity would result from the conjugates compared to the gold(iii) porphyrin complex alone.

**Fig. 1:**
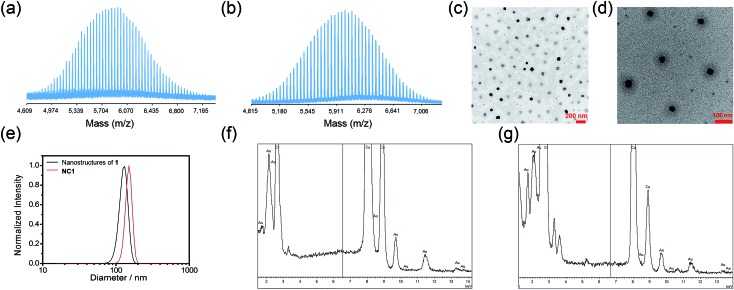
MALDI-TOF-MS of (a) **1** and (b) **2**, using α-cyano-4-hydroxycinnamic acid with sodium trifluoroacetate as the matrix. TEM images of (c) nanostructures of **1** and (d) nanocomposites of **1** and **DOX** (**NC1**) in PBS. (e) DLS profiles of nanostructures of **1** and nanocomposite of **1** and **DOX** in PBS. Energy-dispersive X-ray (EDX) spectra of (f) nanostructures of **1** and (g) **NC1**, respectively.

Gold(iii) porphyrin–PEG conjugates **1** and **2** were synthesized by DCC/HOBt coupling reactions of **4** with H_3_CO–PEG_5000_–OH and H_3_CO–PEG_5000_–NH_2_·HCl, respectively. The crude products in dichloromethane were precipitated three times in diethyl ether, and further purified by column chromatography on neutral alumina using dichloromethane–methanol (50 : 1, v/v) as the eluent. The complexes were characterized using ^1^H NMR spectroscopy (Fig. S1 in ESI†) and matrix-assisted laser desorption/ionization time-of-flight mass spectrometry (MALDI-TOF-MS; Fig. 1a and b and S2†). The polydispersity index (PDI) of **1** and **2** determined from MALDI-TOF-MS were both found to be 1.01, indicating the high purity of the conjugates. The parent gold(iii) complexes of the conjugates, **3** and **4**,^50^ and **Au1a** ^51^ were synthesized according to reported methods, and were well characterized by NMR and MS. Details of the synthesis are included in the ESI.†

### Self-assembly in aqueous media

Conjugates **1** and **2** were found to self-assemble into core–shell micelles with diameters of *ca.* 120 and 200 nm, respectively, by adding their respective acetonitrile solutions into phosphate buffered saline (PBS) followed by evaporation of the volatile solvent, as revealed by transmission electron microscopy images (TEM; Fig. 1c and S3a†). Intensity-averaged signals from dynamic light scattering (DLS) experiments of **1** and **2** were found to be 120.8 ± 7.2 nm and 177.1 ± 81.2 nm respectively (Fig. 1e and S3b†), while the zeta potentials of the nanostructures of **1** and **2** were –0.1 ± 1.6 mV and –0.7 ± 4.2 mV (Fig. S3c and S4†). The good agreement of the intensity-averaged signals from the DLS experiments with the diameters of the nanostructures observed in TEM images suggested the DLS signals corresponding to the hydrodynamic diameters of the nanostructures of **1** and **2**. On the other hand, the almost neutral zeta potentials of the nanostructures of **1** and **2** validated the presence of PEG pendant in the shell of the nanostructures. The identity of gold species in the core of the nanostructures was supported by the good contrast of the core in the TEM images (Fig. 1c and S3a†) and energy-dispersive X-ray (EDX) spectra (Fig. 1f).

### *In vitro* cytotoxicity

The *in vitro* cytotoxicity of **1–3** and **Au1a** to human cancer cell lines (HeLa, NCI-H460, HCT116, A2780) and the cisplatin- and doxorubicin-resistant cancer cell lines (A2780cis and A2780adr), as well as normal human colon mucosal epithelial cell line (NCM460), non-tumorigenic immortalized liver cells (MIHA) and normal lung fibroblast cells (CCD-19Lu), was evaluated using a 3-(4,5-dimethyl-2-thiazolyl)-2,5-diphenyltetrazolium bromide (MTT) assay (Table 1; Fig. S5a†). The ester-linked conjugate (**1**), complex **3** and **Au1a** all demonstrated superior *in vitro* cytotoxicity toward cancer cells, as compared to that of cisplatin (**1** is 5.1–76.9 times more cytotoxic than cisplatin). It is worth noting that **1** retained most of its activity in killing drug-resistant cancer cells (A2780cis and A2780adr), while **Au1a**, **DOX** and cisplatin were found to show significantly lower cytotoxicity against A2780adr, as compared to the non-resistant A2780 cells (Table S1†). For non-tumorigenic cells, **3** and **Au1a** showed IC_50_ in submicromolar range, which is consistent with the reported high toxicity of gold(iii) porphyrin complexes toward normal cells.^6,7^ Interestingly, the nanostructures of **1** were found to be relatively less toxic in non-tumorigenic MIHA, CCD-19Lu and NCM460 cells (Table 1), as revealed by the large ratio of IC_50_ in non-tumorigenic cells to that in cancer cells (Tables S2–S4†). The low toxicity of **1** was also found in experiments with non-tumorigenic liver L02 and gliocyte CHEM-5 cells (Table S5†). On the other hand, the conjugate with the amide linkage (**2**) was less cytotoxic to both cancer and non-tumorigenic cells *in vitro*. To understand the origin of the difference in cytotoxicity of **1–3** and **Au1a**, the release profiles of the conjugates in aqueous buffer solutions and in cells, as well as the cellular uptake of the complexes, were investigated.

**Table 1:** *In vitro* cytotoxicity of **1–3**, **Au1a**, **DOX**, nanocomposite of **1** and **DOX** (**NC1**), and cisplatin

Compound	IC_50_ ^*a*^/μM								
	HeLa	NCI-H460	HCT116	A2780	A2780cis	A2780adr	MIHA	NCM460	CCD-19Lu
**1**	1.91 ± 0.48	0.85 ± 0.07	0.39 ± 0.03	0.51 ± 0.09	0.64 ± 0.03	1.51 ± 0.40	14.49 ± 2.55	16.42 ± 2.45	15.22 ± 1.93
**2**	46.48 ± 4.40	24.66 ± 4.62	8.24 ± 0.22	13.42 ± 0.18	47.25 ± 8.62	>120	114.62 ± 17.11	70.96 ± 6.42	>100
**3**	0.82 ± 0.12	0.11 ± 0.01	0.09 ± 0.01	0.23 ± 0.02	0.19 ± 0.03	0.65 ± 0.06	0.74 ± 0.09	0.13 ± 0.01	0.73 ± 0.04
**Au1a**	0.66 ± 0.04	0.23 ± 0.01	0.09 ± 0.02	0.12 ± 0.02	0.18 ± 0.01	1.13 ± 0.15	0.40 ± 0.07	0.07 ± 0.01	0.23 ± 0.04
**NC1**	0.70 ± 0.15	0.41 ± 0.03	0.09 ± 0.01	0.20 ± 0.02	0.47 ± 0.07	0.53 ± 0.11	3.24 ± 0.37	4.59 ± 1.26	—^*b*^
**DOX**	0.57 ± 0.06	0.04 ± 0.01	0.14 ± 0.02	0.05 ± 0.01	0.05 ± 0.01	0.68 ± 0.05	0.10 ± 0.01	1.01 ± 0.12	—^*b*^
Cisplatin	19.83 ± 1.42	65.42 ± 5.06	15.70 ± 2.2	2.59 ± 0.30	8.36 ± 0.05	12.73 ± 1.83	17.56 ± 1.31	12.32 ± 0.87	49.14 ± 5.43

^*a*^ *In vitro* cytotoxicity was determined by MTT assay upon incubation of the live cells with the compounds for 72 h.

^*b*^Not determined.

### Controlled release properties

**Fig. 2:**
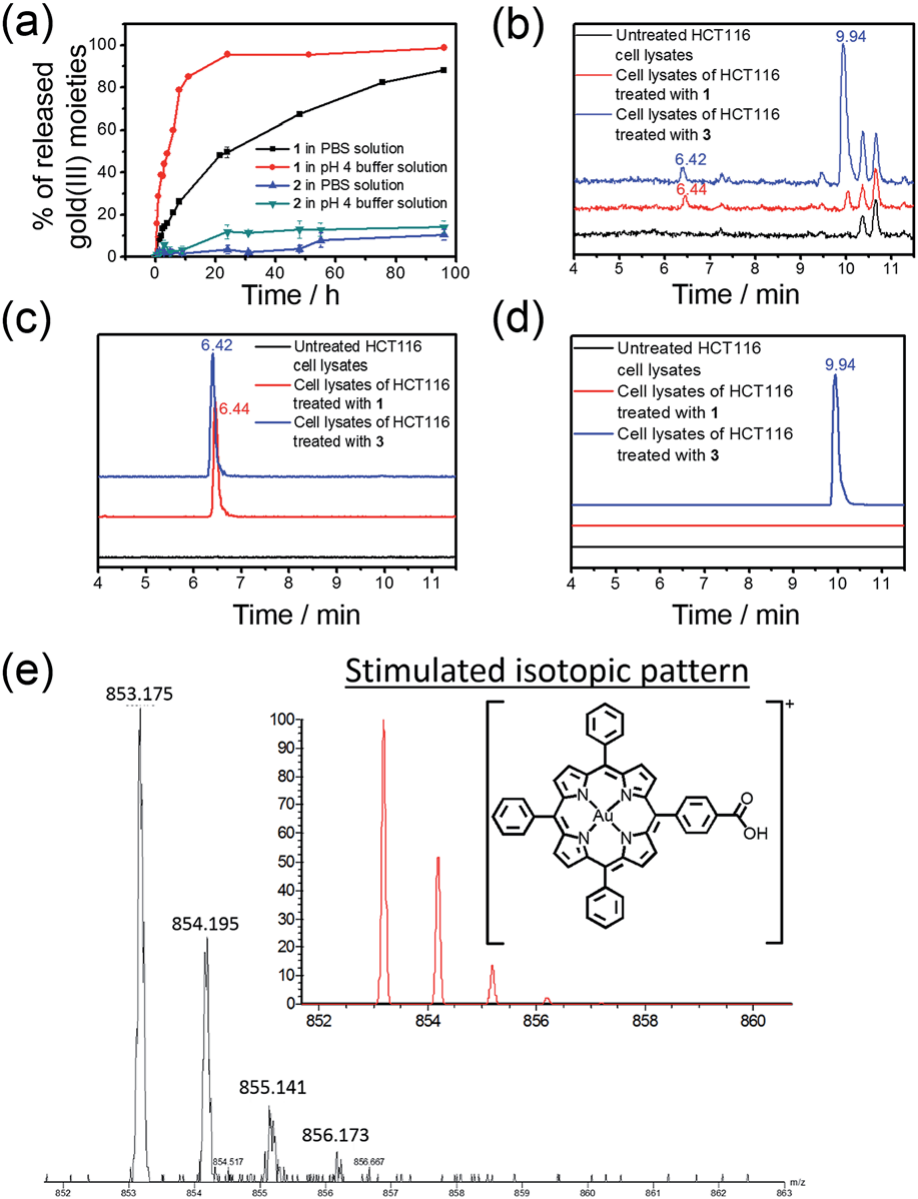
(a) Time-dependent release profiles of **1** and **2** in PBS and/or pH 4 buffer solution as determined by ICP-MS. (b) Total-ion chromatograms of UPLC-QTOF-MS of cell lysates of untreated HCT116 cells and HCT116 cells treated with **1** and **3** (2 μM), respectively, for 24 h. Selected-ion chromatograms at *m*/*z* = (c) 853 and (d) 867, corresponding to the *m*/*z* of [Au(TPP–COOH)]^+^ and [Au(TPP–COOCH_3_)]^+^, respectively. (e) MS recorded at *t* = 6.44 min of the UPLC-QTOF-MS chromatogram of cell lysates of HCT116 cells treated with **1** (2 μM) for 24 h. Inset: simulated isotopic pattern of [Au(TPP–COOH)]^+^.

The rate of the release of the gold(iii) porphyrin moiety from the conjugates in aqueous buffer solutions at different pH values was determined according to the reported methodology.^24,26,27^ Nanostructures of **1** and **2** were dissolved in PBS or sodium acetate buffer (pH 4.0) and then dialyzed against a large volume of PBS or sodium acetate buffer solution in the dark at 37 °C using a 1000 Da cut-off dialysis membrane (details are included in the ESI†). The quantity of the hydrolyzed gold(iii) porphyrin moiety at different time points was determined by inductively coupled plasma mass spectrometry (ICP-MS; Fig. 2a). In PBS, 31% and 60% of **1** were hydrolyzed at 6 and 24 h, respectively, while only 2% and 3% of hydrolyzed product of **2** were found at 5 and 24 h, and more than 89% of **2** remained unhydrolyzed at 96 h. On the other hand, the release of the gold(iii) porphyrin moiety from **1** was found to be faster in more acidic sodium acetate buffer solution (pH 4), with 60% and 96% of the hydrolyzed product found at 6 and 24 h, respectively (Fig. 2a). Faster release was also found upon incubation of **2** in pH 4 buffer solution compared to that in PBS, but the amount was still much smaller than that found in the study of **1**. As the only difference between **1** and **2** is the chemical structure of the linkage for PEG conjugation, the faster release of the gold(iii) porphyrin moiety from **1** in PBS than that from **2** should be attributed to the higher tendency of the ester bond in **1** to undergo hydrolytic cleavage than the amide bond in **2**.^52^ The faster release from **1** in more acidic buffer solution may be ascribed to acid-catalyzed hydrolysis of the ester bond in **1**,^49^ resulting in almost complete cleavage of the PEG pendant after 24 h. ^1^H NMR experiments further supported the hydrolysis of ester linkage of **1** in acidic solution (pH 4), as indicated by the disappearance of peaks from protons of –COOC***H***_2_C***H***_2_– at *δ* = 3.98 and 4.67 ppm after 24 h incubation (Fig. S6†). On the other hand, no observable change in the NMR spectra of **2** was found after incubation in pH 4 or 7.4 solution for 24 h (Fig. S6†), suggesting the high stability of the amide linkage.

Hydrolysis of the gold(iii) complexes in live cells was examined by ultra-performance liquid chromatography coupled quadrupole-time-of-flight mass spectrometry (UPLC-QTOF-MS). Colon cancer cells (HCT116) were treated with **1**, **2** and **3** (2 μM) for 24 h, harvested and lysed in an acetonitrile–water solution mixture (3 : 1, v/v) prior to UPLC-QTOF-MS analysis. The total-ion chromatogram of cell lysates of HCT116 treated with **1** showed a peak with retention time of 6.44 min (Fig. 2b) and *m*/*z* = 853 (Fig. 2c). The mass spectrum of this peak showed a very similar isotopic pattern as that of [Au(TPP–COOH)]^+^ (Fig. 2e). It is noteworthy that this peak was not found in the total-ion chromatogram of **1** in acetonitrile solution, while **4** in acetonitrile solution showed a peak with almost the same retention time (6.39 min) in its total-ion chromatogram (Fig. S7†). Therefore, the peak is attributable to the major metabolite of **1** formed in HCT116 cells. As the peak showed almost the same retention time as the peak of **4** in acetonitrile solution (Fig. S7†) and its mass spectrum revealed a very similar isotopic pattern as that of [Au(TPP–COOH)]^+^ (Fig. 2e), it should originate from the chemical species [Au(TPP–COOH)]^+^, suggesting the hydrolysis of the ester bond of **1** in HCT116 cells to form [Au(TPP–COOH)]^+^. Similarly, hydrolysis of the ester bond was also observed in the study of **3**, which showed the emergence of a peak with a retention time of 6.42 min in the total-ion chromatogram (Fig. 2c), corresponding to [Au(TPP–COOH)]^+^ as supported by its mass spectrum (Fig. S8†). For **2** which has an amide linkage, no release of [Au(TPP–COOH)]^+^ can be found from the treated HCT116 cells (24 h) by UPLC-QTOF-MS analysis (Fig. S9†). This should be attributable to the higher stability of the amide bond *versus* the ester bond *in vitro*.

### Cellular uptake experiments

**Fig. 3:**
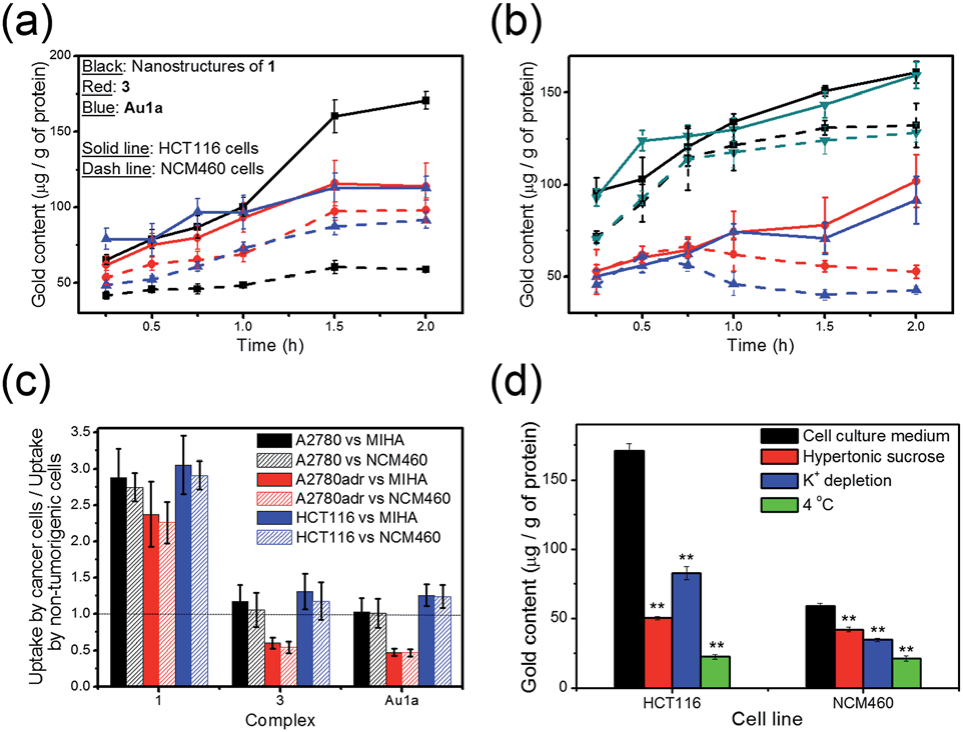
(a) Uptake of **1**, **3** or **Au1a** (all at 2 μM), in terms of the gold content determined by ICP-MS, into HCT116 and NCM460 cells, respectively, after incubation at 37 °C for the indicated time intervals. (b) Uptake of (black) **1**, (red) **3**, (blue) **Au1a** or (dark cyan) nanocomposites of **1** and **DOX**, **NC1**, ([**1**] = 2 μM) into (solid line) A2780 and (dashed line) A2780adr cells, respectively, after incubation at 37 °C for the indicated time intervals. (c) Bar chart showing the ratio of uptake of the complexes (2 μM) into cancer and non-tumorigenic cells after incubation for 2 h at 37 °C. (d) Uptake of **1** into HCT116 and NCM460 cells after incubation for 2 h in (black) cell culture medium with 10 vol% fetal bovine serum at 37 °C, (red) cell culture medium with 10 vol% fetal bovine serum and 0.45 M sucrose at 37 °C, (blue) aqueous buffer solution (140 mM NaCl, 20 mM HEPES, 1 mM CaCl_2_, 1 mM MgCl_2_ and 1 g L^–1^ d-glucose, pH 7.4) at 37 °C and (green) cell culture medium with 10 vol% fetal bovine serum at 4 °C. All the data are shown as mean ± SEM from three independent experiments. ** denotes *p* < 0.01 *vs.* experiment with cell culture medium.

The uptake of **1** and **3**, and **Au1a** by cancer cells and non-tumorigenic cells was examined in terms of the gold content in the cell lysates using ICP-MS. In contrast to the gold(iii) complexes without PEG conjugation, *i.e.* **3** and **Au1a**, the gold(iii) porphyrin–PEG conjugate **1** was found to show faster cellular uptake into a variety of cancer cells, such as colorectal carcinoma (HCT116), human ovarian carcinoma (A2780), and its drug-resistant variant (A2780adr; Fig. 3a–c). Interestingly, the uptake of **1** into non-tumorigenic colon and liver cells (NCM460 and MIHA respectively) was found to be lower than that of **3** and **Au1a** (Fig. 3a and S10†), resulting in significantly higher uptake into cancer cells compared to that into non-tumorigenic cells (Fig. 3c). On the other hand, the uptake of **1** into cancer cells was inhibited by lowered temperature, depletion of K^+^ concentration and hypertonic sucrose concentration, unlike the uptake of **3** which was not significantly affected by these parameters (Fig. 3d, S11 and S12†). This suggests that the nanostructures of **1** likely entered live cells by energy-dependent clathrin-mediated endocytosis, which is in accordance with reported mechanisms of cellular uptake of nanostructures.^30,32^ Due to the generally higher metabolic rate of cancer cells than normal cells, **1** showed faster accumulation in cancer cells compared with non-tumorigenic cells (Fig. 3a–c). It is worth noting that **1** exhibited an increasing concentration in both A2780 and A2780adr cells with time, while **3** and **Au1a** revealed decreases in the amount in A2780adr cells after incubation for 0.75 and 0.5 h, respectively (Fig. 3b). The decrease of **3** and **Au1a** in A2780adr cells were likely due to the P-glycoprotein-mediated drug-resistance of A2780adr cells,^53^ while the accumulation of **1** in A2780adr cells suggested that the nanostructures of **1** could overcome the efflux pump-mediated drug-resistance in these cells.

### Higher *in vitro* cytotoxicity of **1** in cancer cells compared to non-tumorigenic cells

The controlled release property of **1** (Fig. 2 and S7†) and its higher cellular uptake by cancer cells (Fig. 3 and S10–S12†) likely account for the observed higher *in vitro* cytotoxicity of **1** against cancer cells than that against non-tumorigenic cells (Tables S2–S4†). Presumably owing to the generally higher metabolic rate of cancer cells, the nanostructures of **1** accumulated more efficiently in cancer cells than in non-tumorigenic cells. In addition, the acidic character of cancer cells^34^ would favor the hydrolysis of **1**, leading to a higher rate of release of the anti-cancer gold(iii) porphyrin moiety within cancer cells. On the other hand, in the less acidic non-tumorigenic cells, **1** is likely to be hydrolyzed less readily, and the unhydrolyzed gold(iii) porphyrin–PEG conjugates should possess low *in vitro* cytotoxicity, as evidenced by MTT assays of cells treated with **2**, which has an amide linkage with a low tendency for hydrolysis. The latter is attributed to the steric bulk of PEG prohibiting interactions of the gold(iii) porphyrin moiety with its molecular target(s).

For the gold(iii) porphyrin complexes without PEG conjugation, *i.e.* **3** and **Au1a**, they demonstrated high *in vitro* cytotoxicity to both cancer and non-tumorigenic cells (Table 1 and S2–S4†). This may be accounted for by their similar cellular uptake rates into cancer and non-tumorigenic cells (Fig. 3a–c and S10†), resulting in the non-selective killing of cancer and non-tumorigenic cells.

To gain more insight into the higher cytotoxicity of **1** to cancer cells, time-dependent assays on the induction of apoptosis of HCT116 (colon cancer) and NCM460 (normal colon) cells, by **1** and **Au1a** were conducted using CellEvent™ Caspase-3/7 Green ReadyProbes® (Caspase-3/7 Green) Reagent. The Caspase-3/7 Green Reagent is non-luminescent in live cells, but shows strong green fluorescence upon cellular activation of Caspase-3/7, which is an early indicator of apoptosis.^54^ Induction of apoptosis was apparent in both HCT116 and NCM460 cells treated with **Au1a** (2 μM), at 12 and 20 h, respectively (Fig. S13b†), while **1** (2 μM) only induced apoptosis in HCT116 cells after incubation for 15 h and no significant apoptosis of NCM460 cells was found after treatment with **1**, as revealed by the small number of cells showing green luminescence and the cell morphology in the bright-field microscopy images (Fig. S13b†). Flow cytometric analysis of HCT116 cells treated with **1** for 24 and 36 h revealed that 35.83 and 43.83% (**Q4** region of Fig. S14 and S15†; corrected by deduction of the percentage of apoptotic cells found in the negative control) of total population of cells were apoptotic, while 1.96 and 10.6% of total population exhibited late apoptotic events (**Q2** region; corrected). In contrast, **1** was less toxic to NCM460 cells, with 100.9 and 90.14% viability (**Q3** region; corrected) found after incubation for 24 and 36 h respectively. **Au1a** (2 μM) induced a large number of apoptotic events in both HCT116 and NCM460 cells (Fig. S14 and S15†). Smaller populations of viable NCM460 cells (61.14 and 56.64% [corrected], after incubation for 24 and 36 h, respectively) were found, as compared to those treated with **1** for the same time intervals.

**Fig. 4:**
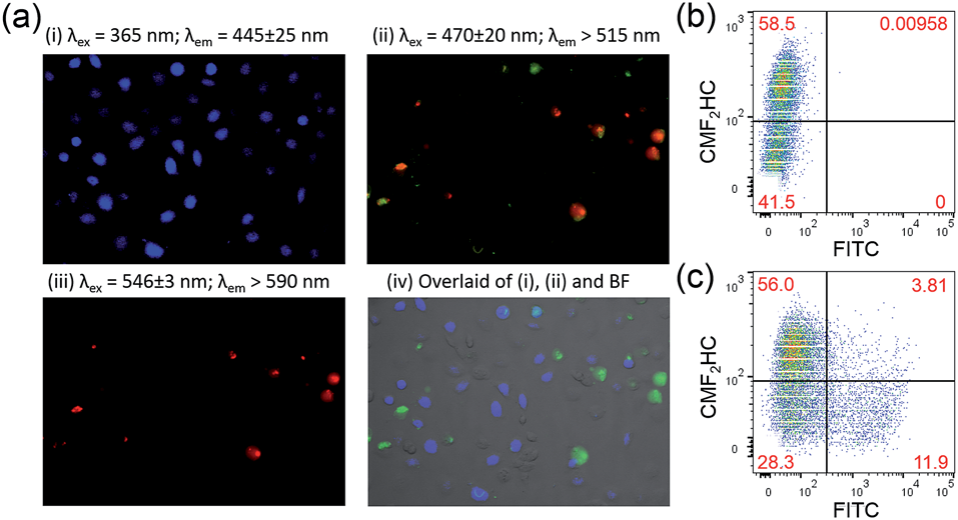
(a) Fluorescence microscopy images of co-cultured HCT116 and NCM460 cells stained with FITC-Annexin V and propidium iodide after incubation with **1** (2 μM) for 24 h. The NCM460 cells were pre-treated with blue-emissive CMF_2_HC dye for differentiation from HCT116 cells. The overlaid image was prepared using ImageJ, with apoptotic cells in green. Analysis by flow cytometry of the co-culture model of NCM460 cells, which were pre-treated by CMF_2_HC dye, and HCT116 cells after incubation with **1** (2 μM) for 24 h and (b) without and (c) with FITC-Annexin V staining.

As **1** was found to show faster cellular uptake into HCT116 cells than **Au1a** (Fig. 3a and c), the induction of apoptosis by **1** after a longer incubation time indicated a time lag for **1** to induce Caspase-3/7 activation in HCT116 cells, when compared to **Au1a** (Fig. 4a). This can be explained by the time required for **1** to undergo hydrolytic cleavage of the ester bond before exhibiting its anti-cancer properties. Nonetheless, both **1** and **Au1a** (2 μM) could induce apoptosis in a large population of HCT116 cells after 48 h (Fig. S13b†), suggesting the readiness of hydrolysis of **1** in HCT116 cells and the effective killing of these colon cancer cells by **1**. For normal colon NCM460 cells, **Au1a** (2 μM) induced their apoptosis after incubation for 20 h, which was slightly longer than that found in HCT116 cells, partly owing to its slower uptake into NCM460 cells (Fig. 3a and c). The lack of significant activation of Caspase-3/7 in NCM460 cells after incubation with **1** can be rationalized by the relatively slow uptake of **1** (Fig. 3a and c), in addition to likely slow hydrolytic cleavage of the PEG pendant of **1** in NCM460 cells.

The induction of apoptosis of HCT116 and NCM460 cells by **1** was further studied using FITC-Annexin V staining. HCT116 cells incubated with **1** and **Au1a** (2 μM), respectively, for 24 h were found to show green fluorescence from FITC-Annexin V, as well as red fluorescence from propidium iodide which is an indicator of late apoptotic or necrotic cells (Fig. S16†).^54^ NCM460 cells treated with **Au1a** also exhibited green and red fluorescence from FITC-Annexin V and propidium iodide respectively (Fig. S16b†), while only a small population of NCM460 cells revealed green fluorescence after incubation with **1** for the same time interval (Fig. S16a†). The quantity of apoptotic cells stained by FITC-Annexin V after treatment with **1** or **Au1a** was also investigated by flow cytometry. After accounting for the background green fluorescence from the negative controls of HCT116 and NCM460 cells (Fig. S17c†), the percent of apoptotic HCT116 cells after incubation with **1** and **Au1a** for 24 h were found to be 22.5 and 39.6%, respectively, while 2.4 and 39.3% of NCM460 cells were found to undergo apoptosis after treatment with **1** and **Au1a** respectively (Fig. S17a and b†). This suggests that **1** was almost non-toxic to normal colon NCM460 cells upon incubation for 24 h, but remained active in inducing apoptotic events in colon cancer HCT116 cells.

To further verify the ability of **1** to selectively induce apoptosis of HCT116 cells over NCM460 cells, HCT116 and NCM460 cells were co-cultured and incubated with **1** for 24 h, and then stained with FITC-Annexin V and propidium iodide. In order to differentiate the two different cell lines, NCM460 cells were pre-treated with blue-emissive CMF_2_HC dye prior to its co-culture with HCT116 cells. Flow cytometric analysis revealed successful differentiation of the two cell lines based on the detection of the blue fluorescence (58.5 and 41.5%; Fig. 4b). In a parallel experiment, co-cultured cells treated with **1** for 24 h and stained with FITC-Annexin V and propidium iodide were imaged by fluorescence microscopy (Fig. 4a). Green and red fluorescence were primarily observed in cells without showing blue luminescence, *i.e.* HCT116 cells. By flow cytometry (Fig. 4c), 20.6 and 1.87% of HCT116 and NCM460 cells, respectively (corrected based on a negative control experiment; Fig. S18†), were found to undergo apoptosis upon incubation with **1** (2 μM) for 24 h. The results of both the fluorescence microscopy and flow cytometry support the high selectivity of **1** on killing colon cancer cells in the co-culture model of cancer and normal cells.

**Fig. 5:**
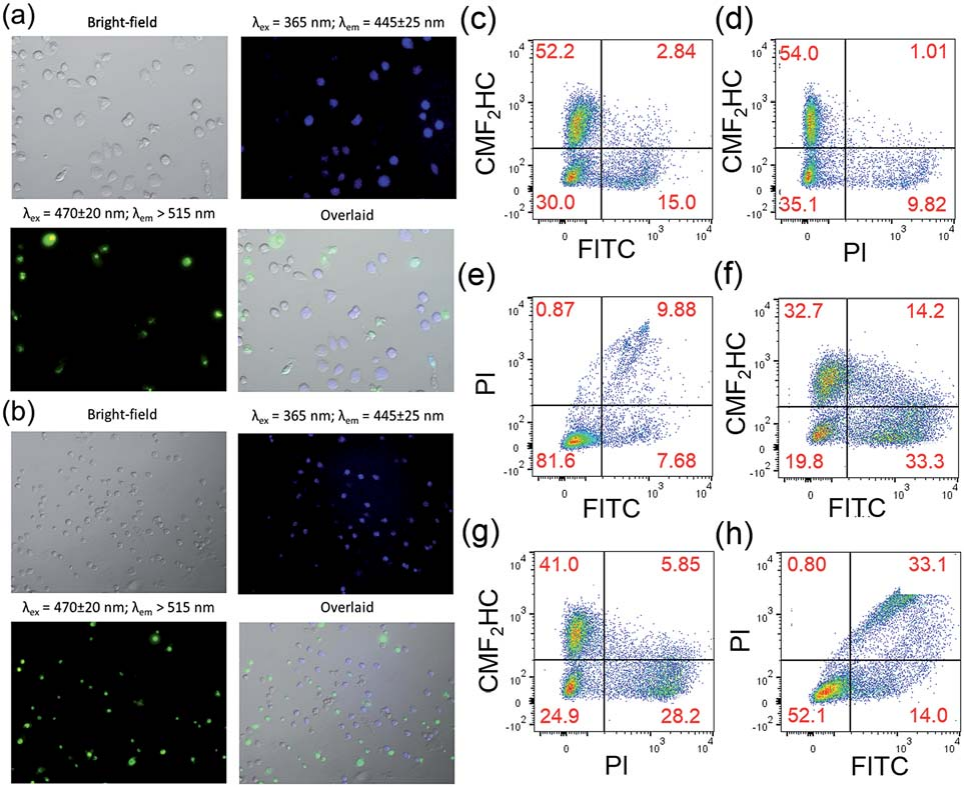
Fluorescence microscopy images of co-cultured HCT116 and NCM460 cells stained with CellEvent™ Caspase-3/7 Green ReadyProbes Reagent after incubation with **1** (2 μM) for (a) 24 h and (b) 36 h, respectively. Flow cytometric analysis of the co-culture model stained with CellEvent™ Caspase-3/7 Green Ready Detection Reagent (2.5 μM) after incubation with (c–e) **1** (2 μM) and (f–h) **Au1a** (2 μM) for 24 h. The NCM460 cells were pre-treated with blue-emissive CMF_2_HC dye for differentiation from HCT116 cells. The overlaid images were prepared using ImageJ.

Staining of the co-culture of HCT116 and NCM460 cells treated with **1** and **Au1a** (2 μM), respectively, with the Caspase-3/7 Green Reagent showed similar findings as those of the experiments with FITC-Annexin V staining. Fluorescence microscopy images of the co-culture model treated with **1** and stained with Caspase-3/7 Green Reagent revealed strong green fluorescence from HCT116 cells, while only a small population of cells with blue luminescence, *i.e.* NCM460 cells, displayed green fluorescence (Fig. 5a and b). On the other hand, a larger population of NCM460 cells in the co-culture model showed green fluorescence after incubation with **Au1a** (2 μM) for 24 and 36 h (Fig. S19†). By flow cytometric analysis (Fig. 5c–e), the population percentages of apoptotic HCT116 and NCM460 cells in the co-culture model after treatment with **1** and **Au1a** for 24 h were found to be 25.3 and 4.1%, and 54.6 and 29.3% (corrected based on a negative control experiment) respectively. Together the data indicate a high cytotoxicity of **Au1a** to both HCT116 and NCM460 cells, while **1** exerts a selective induction of apoptosis in HCT116 cells over NCM460 cells in the co-culture.

To ensure that the results were not due to the effect of CMF_2_HC dye on the co-culture, another co-culture of the two cell lines was prepared, with the HCT116 cells pre-treated with CMF_2_HC dye and hence they would show blue fluorescence upon excitation at 365 nm (Fig. S20†). This co-culture model treated with **1** for 24 h had 25.04 and 5.01% (**Q2** and **Q3** regions of Fig. S21a†; corrected) of HCT116 and NCM460 cells, respectively, undergoing apoptosis as determined by Caspase-3/7 Green Reagent staining and flow cytometric analysis (Fig. S21†). This confirms the preferential induction of apoptosis by **1** of HCT116 cells over NCM460 cells in the co-culture model, regardless of the effect, if any, of the blue emissive CMF_2_HC dye.

### Promising *in vivo* anti-tumor activities with minimal systemic toxicity

**Fig. 6:**
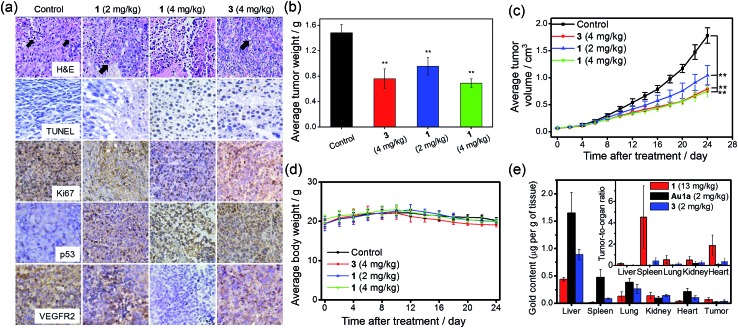
Tumor growth inhibition in nude mice bearing HCT116 xenografts after treatment with **1** or **3**. (a) Immunohistochemical analysis of tumor sections of nude mice (*n* = 8) after intravenous injections with solvent control, different doses of **1** or **3** every two days for 24 days. The black arrows denote the nuclear atypia and mitosis in tumor sections. (b) The tumor weight of nude mice (*n* = 8) treated with solvent control, different doses of **1** or **3**, measured at 24 days post treatment. (c) Changes in tumor volume and (d) body weight, respectively, in mice (*n* = 8) after treatment with solvent control, different doses of **1** or **3**. The control group received an equal volume of PBS only. ** denotes *p* < 0.01 *vs.* control. (e) Biodistribution of gold compounds in different organs of nude mice bearing HCT116 xenografts after single-dose intravenous injection with **1** (13 mg kg^–1^; *n* = 5), **Au1a** (2 mg kg^–1^; *n* = 4) and **3** (2 mg kg^–1^, *n* = 4) at 24 h post-treatment. The gold contents in homogenized tissues were quantified by ICP-MS. Solvent control was used as background for calculations. Inset shows the ratios of gold content in tumor to that in other organs.

**Fig. 7:**
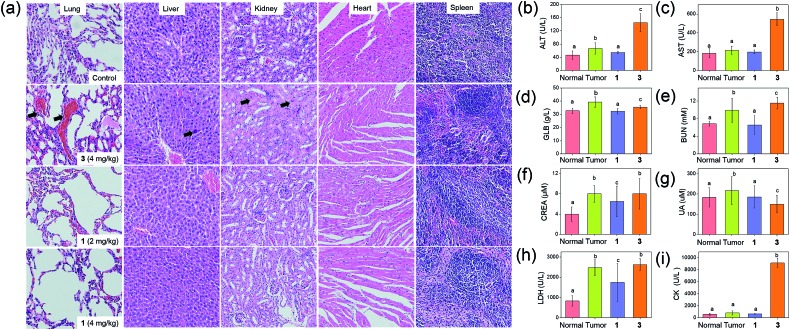
Evaluation of systemic toxicity in nude mice bearing HCT116 xenografts after treatment with **1** or **3**. (a) H & E staining of lung, liver, kidney, heart and spleen sections from HCT116 xenografted nude mice (*n* = 8) at 24 days post-treatment. The black arrows in lung, liver and kidney sections denote sites of pulmonary hemorrhage, apoptotic cells and swollen epithelial cells, respectively. Blood biochemistry analysis of (b) ALT, (c) AST, (d) GLB, (e) BUN, (f) CREA, (g) UA, (h) LDH and (i) CK in the mice (*n* = 8) at 24 days post-treatment. Bars with different characters were statistically significant at *p* < 0.05 level.

The anti-tumor experiments using mice were approved by the animal ethics committees of Jinan University and The University of Hong Kong. Treatment of nude mice bearing HCT116 xenografts with **1** (2 or 4 mg kg^–1^) for 24 days through intravenous injection resulted in a significant reduction of tumor weight (35 and 53%, respectively; *p* < 0.01, *n* = 8; Fig. 6b) and tumor volume (41 and 58% respectively; *p* < 0.01, *n* = 8; Fig. 6c), with no mouse death or significant loss in body weight (Fig. 6d). Histopathological analysis of the tissues of treated mice revealed that no significant anomaly was observed in lung, liver, kidney, heart and spleen after treatment with **1** (Fig. 7a). The low systemic toxicity of **1** was further supported by the blood biochemistry of nude mice after treatment with **1** (4 mg kg^–1^; Fig. 7b–i); plasma levels of several organ damage indicators including alanine transaminase (ALT), globulin (GLB), blood urea nitrogen (BUN) and creatine kinase (CK) of the treated mice were lower than those of the untreated mice bearing HCT116 xenografts (*p* < 0.05; Fig. 7b, d, e and g), and fell within the statistically relevant range of those of mice without the xenograft. In addition, [Au(TPP–COOH)]^+^ was found in the UPLC-QTOF-MS of tumor tissues of mice treated by **1**, supporting the propensity for hydrolysis of **1** *in vivo* (Fig. S22†).

On the other hand, intravenous injection of **3** (4 mg kg^–1^) to nude mice bearing HCT116 xenografts resulted in a significant reduction of tumor weight and tumor volume (49 and 56% respectively; *p* < 0.01, *n* = 8; Fig. 6b and c). Yet, higher concentration of **3** was required to achieve similar inhibition of tumor growth as found in the treatment with **1** (4.43 mmol kg^–1^ *vs.* 0.68 mmol kg^–1^), suggesting the more potent anti-tumor effect of **1**. Immunohistochemical analyses of tumor sections further supported the superior anti-tumor activities of **1** over that of **3** (Fig. 6a). Effective elimination of nuclear atypia and mitosis were observed after treatment of the mice with **1** (4 mg kg^–1^), but not for the treatment with **3** (4 mg kg^–1^). This, together with the more significant inhibition of the expression of Ki67 and VEGFR2, enhanced expression of p53 and elevated DNA fragmentation (from TUNEL staining) by **1**, demonstrates that **1** can effectively decrease cancer cell proliferation and induce cancer cell apoptosis *in vivo*. More importantly, in contrast to the low systemic toxicity of **1**, histopathological analysis showed damage in normal lung, liver and kidney tissues after treatment with **3** (4 mg kg^–1^; Fig. 7a). Significant pulmonary hemorrhage in the alveoli and a thickening of alveolar walls were found. Also, apoptotic hepatocytes and swollen renal epithelial cells were found, leading to subsequent shrinkage of kidney tubules. The blood biochemistry of mice treated with **3** indicated significant damage in the lung, liver and kidney of the mice (Fig. 7b–i). The lower systemic toxicity of **1** can be explained by the lower accumulation of **1** in the organs of mice treated at its effective dosage, as compared to that of **3** and **Au1a** (Fig. S23†). *In vivo* biodistribution study of HCT116 xenografted nude mice subjected to single-dose intravenous injections of equal molar amount of gold compounds at 24 h post-treatment revealed higher accumulation of **1** (determined by ICP-MS analysis) in tumor compared to mice treated with **Au1a** (by 3.3-fold) or **3** (by 2.0-fold) (Fig. 6e). As lower gold contents were found in liver, spleen, lung, kidney and heart of **1**-treated mice, **1** showed significantly higher tumor-to-organ ratio than **Au1a** or **3** (Fig. 6e). This can be attributable to the nanostructures of **1**, which may lead to *in vivo* tumor accumulation by the EPR effect.^55^

**Fig. 8:**
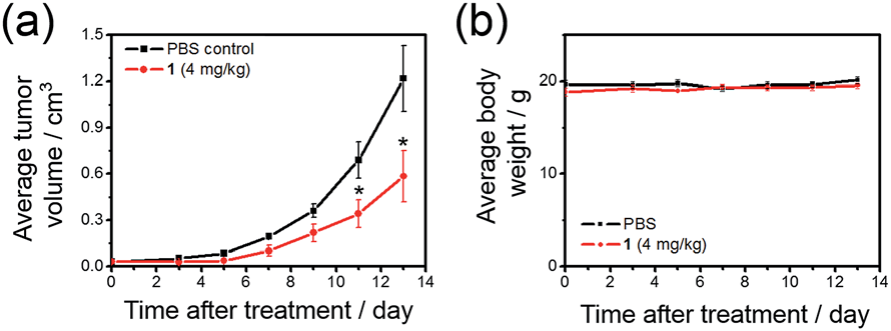
Tumor growth inhibition in nude mice bearing cisplatin-resistant A2780cis xenografts. Change in (a) tumor volume and (b) body weight, respectively, in nude mice treated with **1** (4 mg kg^–1^) or solvent control over time. * denotes *p* < 0.05 *vs.* control. Data are shown as mean ± SD (*n* = 5).

To further investigate the ability of **1** to treat drug-resistant tumors that should be of great interest in clinical application, *in vivo* antitumor experiments on nude mice bearing cisplatin-resistant A2780cis xenografts with **1** were conducted. Over 52% reduction (*p* < 0.05) of tumor volume was found after treatment of mice with **1** (4 mg kg^–1^, intravenous injections for every 2–3 days for 13 days; *n* = 5; Fig. 8a), with no mouse death or significant loss in body weight (Fig. 8b). This suggests that **1** can be useful for the development of anti-cancer agents for treating drug-resistant tumors.

### Encapsulation of doxorubicin for chemotherapeutic co-delivery

Doxorubicin (**DOX**), which has been clinically approved for the treatment of ovarian cancer and multiple myeloma, elicits severe cardiotoxicity when it is administered alone.^29,30^ Also, resistance to doxorubicin, owing to P-glycoprotein efflux pump, has developed in some types of ovarian cancer cells, such as those of the A2780adr line.^53^ In view of the interesting nanostructures formed by **1**, its ability to overcome efflux pump-mediated drug resistance of A2780adr cells as suggested by the cellular uptake experiments (Fig. 3b) and the relatively low toxicity toward non-tumorigenic cells than that toward cancer cells, we were inspired to encapsulate doxorubicin in nanostructures of **1** in an effort to formulate a more potent therapeutic.

Encapsulation of **DOX** by nanostructures of **1** was carried out by co-assembly of **1** and **DOX** through addition of acetonitrile solution of **1** (2.5 mg) and **DOX** (0.18 mg) into PBS, followed by evaporation of the volatile solvent (Scheme 1c). Nanostructures of *ca*. 120 nm were observed in the TEM image of nanocomposites of **1** and **DOX** (**NC1**; Fig. 1d). Profiles of DLS experiments and zeta potential measurements indicated that the nanostructures of **NC1** have hydrodynamic radii of 122.1 ± 13.4 nm and zeta potentials of –2.5 ± 1.6 mV respectively, similar to those of the nanostructures of **1** (Fig. 1e and S4†). The gold content in **NC1** was confirmed by EDX (Fig. 1g). Due to significant overlapping of the UV-vis absorption spectra of **1** and **DOX** which hindered the quantification of **1** and **DOX** in the nanocomposites, the concentration of **1** in **NC1** was determined by ICP-MS experiments. On the other hand, **DOX** concentration in **NC1** was measured by UPLC coupled with photodiode array for detection using daunorubicin hydrochloride as an internal standard (Fig. S24†). From the results of the two experiments, the mole ratio of **1** to **DOX** in **NC1**, as well as the encapsulation efficiency of **DOX** by **1**, were found to be 7 : 1 and 16.5%, respectively (Table 2).

**Table 2:** Physiochemical properties of nanocomposites of **1** and **DOX**

	Feed ratio ([**1**] : [**DOX**])	Found mole ratio of **1** ^*a*^ to **DOX** ^*b*^	Hydrodynamic diameter/nm	Zeta potential/mV	%^*c*^
**NC1**	1.41	7 : 1	122.1 ± 13.4	–2.5 ± 1.6	16.5
**NC2**	0.56	2.2 : 1	139.6 ± 20.9	–0.8 ± 4.2	11.2
**NC3**	0.30	1.3 : 1	133.5 ± 19.2	–1.2 ± 4.2	9.4

^*a*^The concentration of **1** in the nanocomposites was determined by ICP-MS.

^*b*^The concentration of **DOX** in the nanocomposites was determined by UPLC coupled with photodiode array detector using daunorubicin hydrochloride (3.6 μM) as the internal standard.

^*c*^Encapsulation efficiency.

An increase in the feed ratio of **DOX** to **1** was found to increase the loading of **DOX** into the nanocomposites (Table 2), as indicated by UPLC analysis. However, a decrease in encapsulation efficiency was also found with increasing feed ratio of **DOX** to **1** (Table 2). For nanocomposites with different **DOX** loadings, they were found to show similar size and surface charge to each other, as reflected by the DLS and zeta potential measurements (Table 2).

### Synergistic effect on killing cancer cells and lower toxicity toward non-tumorigenic cells by **NC1**

**NC1** was found to be more potent in killing cancer cells than either nanostructures of **1** or **DOX** (Table 1; Fig. S25†). For example, according to Caspase-3/7 Green Reagent assays, **NC1** ([**1**] = 3.5 μM and [**DOX**] = 0.5 μM) induced apoptosis in HCT116 cells after incubation for 18 h, while a 30 h incubation was required for significant induction of apoptosis of HCT116 cells by **DOX** alone (Fig. S26a†). The *in vitro* cytotoxicity of **NC1** was also compared to that of a mixture of nanostructures of **1** and **DOX**. Such a combination exhibited higher cytotoxicity to HeLa cells than either individual component, but was relatively less effective compared to **NC1** (Table 3). The observed cytotoxicity was further evaluated by combination index (CI),^56^ which extrapolates the effect of drug combinations. Based on the calculated CI value at IC_50_ of the drug combinations by CompuSyn,^56^ both **NC1** and the mixture of nanostructures of **1** and **DOX** showed synergism on killing HeLa cells, with the former treatment being more potent. The synergistic effect can originate from different molecular targets and/or mechanism of action of **DOX** from those of gold(iii) porphyrin complexes. DNA is expected to be the primary target of **DOX**,^57^ while gold(iii) porphyrin complexes have been found to cause depletion of mitochondrial membrane potential and induce apoptosis by both caspase-dependent and caspase-independent mitochondrial death pathways.^6^ These different modes of action would allow a supra-additive effect on killing HeLa cells by co-delivery. For the observation of stronger synergism in **NC1** than the mixture of **1** and **DOX**, this can be attributed to the synchronized delivery of the two chemotherapeutics to HeLa cells from **NC1**.^58^

**Table 3:** *In vitro* cytotoxicity of drug combinations of **1** and **DOX** upon incubation with HeLa, A2780adr and MIHA cells, respectively, for 72 h

Cell lines	Compounds/drug combinations	**1** at IC_50_/μM	**DOX** at IC_50_/μM	Total dose at IC_50_/μM	CI^*a*^
HeLa	**1**	1.91	—	1.91	—
	**DOX** alone	—	0.57	0.57	—
	Co-incubation ([**1**] : [**DOX**] = 7 : 1)	0.74	0.11	0.85	0.57
	**NC1**	0.62	0.09	0.71	0.48
A2780adr	**1**	1.51	—	1.51	—
	**DOX** alone	—	0.68	0.68	—
	Co-incubation ([**1**] : [**DOX**] = 7 : 1)	0.85	0.12	0.97	0.75
	**NC1**	0.53	0.08	0.61	0.47
MIHA	**1**	14.49	—	14.49	—
	**DOX** alone	—	0.10	0.10	—
	Co-incubation ([**1**] : [**DOX**] = 7 : 1)	0.57	0.08	0.65	0.86
	**NC1**	3.24	0.46	3.80	4.87

^*a*^Combination index determined by CompuSyn.

It is noteworthy that **NC1** was relatively less toxic to non-tumorigenic cells than cancer cells (Fig. S26b†; Tables 1 and 3; Tables S6 and S7†). This can be rationalized by slower uptake of nanostructures of **1** into non-tumorigenic cells (Fig. 3), and hence likely reduced the uptake of **DOX** in the form of **NC1**, as compared to the administration of **DOX** alone, resulting in less effective killing of MIHA cells and the apparent “strong antagonism” (CI = 4.9; Table 3).

### Overcoming efflux pump-mediated drug resistance of A2780adr cells by **NC1**

The uptake of encapsulated **DOX** in the form of **NC1** by A2780adr cells was 60.2 and 82.7 μg per g of proteins after incubation for 14 and 24 h respectively, based on the analysis of cell lysates by UPLC-QTOF-MS (Fig. S26c†). On the other hand, only 33.9 and 27.8 μg of **DOX** per g of proteins were found in lysates of A2780adr cells after incubation with free **DOX** for 14 and 24 h respectively, and these values are 1.8- and 3.0-fold lower than those found in the incubation with **NC1** for the same time intervals (Fig. S26c†). This indicates that a significant increase in **DOX** content in A2780adr cells can be achieved when using **1** as a nanocarrier of the chemotherapeutic, thus overcoming the efflux pump drug-resistance of A2780adr cells.^53^

The *in vitro* cytotoxicity of **NC1** and mixtures of **1** and **DOX** against A2780adr cells was also investigated by MTT assay (Table 3; Fig. S25b†). From the calculated CI values at IC_50_ of the drug combinations, co-incubation of A2780adr cells with **1** and **DOX** (CI = 0.75) resulted in moderate synergism,^56^ while **NC1** showed synergism with significant reduction of effective dosage for killing A2780adr cells (CI = 0.47).^56^ The much higher potency of **NC1** than that of the mixtures of **1** and **DOX** should not simply be attributed to the synchronized delivery of the two chemotherapeutics, as observed in the experiments with HeLa cells (Table 3); in view of the overcoming of efflux pump-mediated drug-resistance by the nanostructures (Fig. 3b and S26c†), the higher accumulation of **DOX** in A2780adr cells from the incubation with **NC1** (Fig. 8b) should be the main reason for effective killing of A2780adr cells.

## Conclusions

Multi-functional gold(iii) porphyrin–PEG conjugates have been synthesized and characterized. The amphiphilic conjugate **1** has been found to undergo self-assembly into nanostructures in aqueous media. Hydrolysis of its ester linkage in acidic buffer solution, live cancer cells or tumor tissues resulted in the release of the anti-cancer gold(iii) porphyrin moiety without the formation of toxic byproducts. This led to a potent *in vitro* cytotoxicity to a panel of cancer cell lines. Together with the self-assembly into nanostructures and hence higher uptake into cancer cells than non-tumorigenic cells, **1** has demonstrated good selectivity in killing cancer cells over non-tumorigenic cells. This was supported by MTT assays, time-dependent fluorescence microscopy imaging and flow cytometry as well as experiments exploiting a co-culture model of cancer and normal cells. Treatment of nude mice bearing HCT116 xenografts with **1** was found to inhibit tumor growth *in vivo*. Histopathological analysis of tissue sections and biochemical assays of blood samples from treated mice suggest a low systemic toxicity of **1**. The *in vivo* antitumor activities and low systemic toxicity can be attributable to the nanostructures of **1**, leading to tumor targeting by EPR effect and hence low accumulation of **1** in organs of treated mice. More importantly, **1** could also inhibit tumor growth in nude mice bearing A2780cis xenografts *in vivo*, demonstrating its potential application for the treatment of cisplatin-resistant tumors. The nanostructures of **1** have been further utilized to encapsulate chemotherapeutic doxorubicin, resulting in synergism on killing HeLa cells, as indicated by a low Combination Index, and lower toxicity to non-tumorigenic cells. Also, the nanocomposites could overcome efflux pump-mediated drug resistance found in A2780adr cells, thus effective killing of the drug-resistant cancer cells can be accomplished by the nanocomposites but not doxorubicin alone. All of these features suggest that the self-assembly of gold(iii) porphyrin–PEG conjugate not only allowed the complex to exhibit high efficacy in killing cancer cells, but also to function as a nanocarrier for drug co-delivery which should be advantageous for anti-cancer treatment. As a number of reported anti-cancer metal complexes are known to contain hydrophobic π-conjugated ligands, the present work opens up a new avenue for the functionalization of hydrophobic anti-cancer metal complexes into amphiphilic metal complexes. Such amphiphilic metal complexes can undergo self-assembly into nanostructures and achieve promising anti-cancer properties with a significant reduction of toxic side effects. Also, the approach described herein provides new insights into the utilization of the self-assembly properties of anti-cancer metal complexes for encapsulation of therapeutics, thus minimizing the need of components for nano-assembly.
