# Supplementary material for: A multi-functional PEGylated gold(iii) compound: potent anti-cancer properties and self-assembly into nanostructures for drug co-delivery
Source: Chem Sci. 2016 Nov 22;8(3):1942–53. doi: 10.1039/c6sc03210a (PMC5384453; doi:10.1039/c6sc03210a)
Supplement: Supplementary file 1 [file SC-008-C6SC03210A-s001.pdf]

Electronic Supplementary Material (ESI) for Chemical Science.
This journal is © The Royal Society of Chemistry 2016

Electronic Supplementary Information

A multi-functional PEGylated gold(III) compound: Potent anti-cancer properties and self-assembly into nanostructures for drug co-delivery

Clive Yik-Sham Chung,[a] Sin-Ki Fung,[a] Ka-Chung Tong,[a] Pui-Ki Wan,[a] Chun-Nam Lok,[a] Yanyu Huang,[b] Tianfeng Chen[b] and Chi-Ming Che[*a]*

[a] State Key Laboratory of Synthetic Chemistry, Department of Chemistry and Chemical Biology Centre, The University of Hong Kong, Pokfulam Road, Hong Kong, China

[b] Department of Chemistry, Jinan University, Guangzhou 510632, China

*To whom correspondence should be addressed. E-mail: cmche@hku.hk

Table of contents

Methods ·········· P.3
References ·········· P.15
Fig. S1 ^{1}H NMR spectra of **1** and **2** in $CDCl_3$ ·········· P.16
Fig. S2 MALDI-TOF MS of $H_3CO–PEG_{5000}–OCH_3$ and $H_3CO–PEG_{5000}–NH_2$•HCl ·········· P.17
Fig. S3 TEM image, DLS profile and profile showing the zeta potential of nanostructures of **2** in PBS solution ·········· P.18
Fig. S4 Profiles showing the zeta potential of nanostructures of **1** and nanocomposite of **1** and **DOX** in PBS solution ·········· P.19
Fig. S5 Profiles showing cell viability upon treatment with different concentrations of nanostructures of **1** and nanocomposite (**NC1**) of **1** and **DOX** for 72 h ·········· P.20
Fig. S6 ^{1}H NMR spectra of **1** and **2** after incubation in pH 4 and 7.4 buffer solutions at 37 ^oC for 24 h ·········· P.21
Fig. S7 Total-ion chromatograms of UPLC-QTOF-MS of **1**, **3** and **4** in acetonitrile solution ·········· P.22
Fig. S8 MS of the UPLC-QTOF-MS chromatogram of cell lysates of HCT116 cells treated with **3** for 24 h ·········· P.23
Fig. S9 Total-ion and selected-ion chromatograms from UPLC-QTOF-MS of cell lysates of untreated HCT116 cells and HCT116 cells treated with **2** for 24 h ·········· P.24
Fig. S10 Cellular uptake of the complexes by MIHA cells after incubation at 37 ^oC for indicated time intervals ·········· P.25
Fig. S11 Cellular uptake of **1** and **3** into HCT116 and NCM460 cells after incubation at 4 ^oC for indicated time intervals ·········· P.26
Fig. S12 Bar chart showing the ratio of cellular uptake of the complexes into HCT116 and NCM460 cells after incubation for 2 h ·········· P.27

Fig. S13 Fluorescence microscopy images of HCT116 cells and NCM460 cells after incubation with CellEvent Caspase-3/7 Green ReadyProbes Reagent and nanostructures of **1** and **Au1a** at different time intervals ···· P.28

Fig. S14 Flow cytometric analysis of the induction of apoptosis of HCT116 and NCM460 cells (incubated with nanostructures of **1**, **Au1a** and solvent control for 24 h) based on CellEvent Caspase-3/7 Green Detection Reagent and propidium iodide staining ···· P.29

Fig. S15 Flow cytometric analysis of HCT116 and NCM460 cells treated with nanostructures of **1**, and NCM460 cells treated with **1** and **Au1a** respectively, for 36 h after FITC-annexin V and propidium iodide staining ···· P.30

Fig. S16 Images of fluorescence microscopy of HCT116 and NCM460 cells stained with FITC-Annexin V and propidium iodide after incubation with **1** and **Au1a** for 24 h ···· P.31

Fig. S17 Analysis of induction of apoptosis of HCT116 and NCM460 cells (incubated with nanostructures of **1**, **Au1a** and solvent control for 24 h) using FITC-Annexin V staining and flow cytometry ···· P.32

Fig. S18 Analysis by flow cytometry of co-culture model of NCM460 cells (pre-treated by CMF_2HC dye) and HCT116 cells after incubation with solvent control for 24 h and subsequent FITC-Annexin V staining ···· P.33

Fig. S19 Fluorescence microscopy images of co-culture model of HCT116 and NCM460 cells stained with CellEvent Caspase-3/7 Green ReadyProbes Reagent after incubation with **Au1a** for 24 h and 36 h ···· P.34

Fig. S20 Flow cytometric analysis of co-culture model of HCT116 (pre-treated by CMF_2HC dye) and NCM460 cells treated with solvent control for 24 h ···· P.35

Fig. S21 Flow cytometric analysis of co-culture model of HCT116 (pre-treated by CMF_2HC dye) and NCM460 cells stained with CellEvent Caspase-3/7 Green Ready Detection Reagent after incubation with **1** and solvent control for 24 h ···· P.36

Fig. S22 Selected-ion chromatograms from UPLC-QTOF-MS at *m/z* = 853, corresponding to the *m/z* of $[Au(TPP–COOH)]^+$, of homogenized tumor tissues of untreated mice and mice treated by **1** ···· P.37

Fig. S23 Biodistribution of gold complexes in nude mice bearing HCT116 xenografts after 14 days of treatment with **1**, **Au1a** and **3** through intravenous injection ···· P.38

Fig. S24 UPLC traces of nanocomposite of **1** and **DOX** (**NC3**) and **DOX** standard, and calibration curve of **DOX**. ···· P.39

Fig. S25 Profiles showing cell viability of HeLa and A2780adr cells upon treatment with different concentrations of **NC1** and **DOX** for 72 h ···· P.40

Fig. S26 Fluorescence microscopy images of HCT116 cells after incubation with CellEvent Caspase-3/7 Green ReadyProbes Reagent, **NC1**, and **DOX** at different time intervals; profile showing cell viability of MIHA cells upon treatment with **NC1** and **DOX** for 72 h; cellular uptake of **NC1** and **DOX** into A2780adr cells after incubation at 37 ^oC for indicated time intervals ···· P.41

Table S1 Difference in *in vitro* cytotoxicity toward A2780 and drug-resistant A2780cis and A2780adr ···· P.42

Table S2 Relative toxicity of **1**–**3** and **Au1a** toward cancer cells and NCM460 cells ···· P.43

Table S3 Relative toxicity of **1**–**3** and **Au1a** toward cancer cells and MIHA cells ···· P.44

Table S4 Relative toxicity of **1**–**3** and **Au1a** toward cancer cells and CCD-19Lu cells ···· P.45

Table S5 *In vitro* cytotoxicity of **1**, **3** and cisplatin toward non-tumorigenic liver L02 and gliocyte CHEM-5 cells ···· P.46

Table S6 Relative toxicity of **DOX** toward cancer cells and MIHA cells through administration of **DOX** alone and administration by **NC1** ···· P.47

Table S7 Relative toxicity of **DOX** toward cancer cells and NCM460 cells through administration of **DOX** alone and administration by **NC1** ···· P.48

Methods

Materials and reagents. Pyrrole, benzaldehyde, 4-formylbenzoic acid, sodium acetate, 4-(dimethylamino)pyridine and 3-(4,5-dimethyl-2-thiazolyl)-2,5-diphenyltetrazolium bromide (MTT) were purchased from Sigma-Aldrich. Propionic acid and trimethylamine were purchased from Acros Organics. Lithium chloride was purchased from Alfa Aesar. *N*,*N'*-Dicyclohexylcarbodiimide (DCC) and *N*-(3-dimethylaminopropyl)-*N'*-ethylcarbodiimide hydrochloride (EDC•HCl) were purchased from AK Scientific. Poly(ethylene glycol) monomethyl ether (PEG_{5000}–OCH_3; M_w = 5,000) and amino-poly(ethylene glycol) monomethyl ether hydrochloride (H_3CO–PEG_{5000}–NH_2•HCl; M_w = 5,000) were purchased from Fluka and JenKem Technology respectively. Doxorubicin hydrochloride and daunorubicin hydrochloride were purchased from Abcam and TCI America respectively. Minimum essential medium (MEM), Dulbecco's modified Eagle's medium (DMEM), RPMI 1640 medium, fetal bovine serum (FBS), phosphate-buffered saline (PBS), trypsin-EDTA, penicillin/streptomycin, CellEvent Caspase-3/7 Green ReadyProbes Reagent, CellEvent Caspase-3/7 Green Detection Reagent and CellTracker™ Blue CMF_2HC Dye were purchased from Invitrogen. Bio-Rad Protein Assay Dye Reagent Concentrate and sodium dodecyl sulfate were purchased from Bio-Rad Laboratories, Inc. Bovine serum albumin (BSA) was purchased from USB Affymetrix. FITC-Annexin V Apoptosis Detection Kit I was purchased from BD Pharmingen. All other reagents were of analytical grade and were used without further purification. MilliQ water was used in all experiments unless otherwise stated.

Cell cultures. Human ovarian cancer cell line (A2780), and the cisplatin- and adriamycin-resistant derivatives, A2780cis and A2780adr respectively, were obtained from Sigma-Aldrich. All the other cell lines were obtained from American Type Culture Collection (ATCC). Human cervical epithelial carcinoma (HeLa) and normal lung fibroblast (CCD-19Lu) were maintained in Eagle's minimum essential medium with 10 vol% fetal bovine serum, L-glutamine (2 mm), and penicillin/streptomycin (100 U/mL). Human non-small lung cancer (NCI-H460), human colorectal carcinoma (HCT116), human ovarian carcinoma (A2780) and normal human colon mucosal epithelial cell line (NCM460) were maintained in RPMI-1640 medium with 10 vol%

fetal bovine serum, L-glutamine (2 mM) and penicillin/streptomycin (100 U/mL). Cisplatin- and adriamycin-resistant derivatives of A2780 (A2780cis and A2780adr) were maintained in RPMI-1640 medium with 10 vol% fetal bovine serum, L-glutamine (2 mM) and penicillin/streptomycin (100 U/mL) as well as cisplatin (1 μM) and doxorubicin (0.1 μM), respectively. Non-tumorigenic immortalized liver cells (MIHA), human normal liver cells (L02) and human normal gliocyte cells (CHEM-5) were maintained in Dulbecco's modified Eagle's medium with 10 vol% fetal bovine serum, L-glutamine (2 mM) and penicillin/streptomycin (100 U/mL). Cells were incubated in 5% CO_2 humidified air, and subcultured when 80% confluence was reached.

Physical measurements and instrumentation. ^{1}H NMR spectra were recorded with a Bruker AVANCE 400 (400 MHz) or DPX-300 (300 MHz) Fourier transform NMR spectrometer at ambient temperature with tetramethylsilane (Me_4Si) as an internal reference. MALDI-TOF mass spectra were recorded on a ABI4800 MALDI TOF/TOF™ Analyzer using α-cyano-4-hydroxycinnamic acid with sodium trifluoroacetate as the matrix. Positive ion electrospray (ESI) mass spectra and ultra-performance liquid chromatography coupled quadrupole-time-of-flight mass spectrometry (UPLC-QTOF-MS) were recorded on a Waters Micromass Q-Tof Premier quadrupole time-of-flight tandem mass spectrometer. Inductively coupled plasma mass spectra (ICP-MS) were recorded on Agilent 7500a inductively coupled plasma mass spectrometer (Agilent Technologies, CA, U.S.A.). Ultra performance liquid chromatography (UPLC) measurements were performed on a Waters ACQUITY UPLC H-Class System equipped with a photodiode array for detection. Elemental analyses were performed on the Carlo Erba 1106 elemental analyzer at the Institute of Chemistry, Chinese Academy of Sciences, Beijing, China. UV-vis absorption spectra were recorded on a Hewlett-Packard 8453 diode array spectrophotometer equipped with a Xenon flash lamp. For MTT and protein assays, the UV-vis absorption was measured using Perkin-Elmer Fusion Reader (Packard BioScience Company). Optical and fluorescence microscopy images of cell culture were taken on a Zeiss Axiovert 200M inverted fluorescence microscopy. Flow cytometry was performed on BD FACSCantoII Analyzer and BD FACSAria SORP. Dynamic light scattering (DLS) and zeta potential measurements were performed

using Zetasizer 3000HSA with internal HeNe laser (λ_0 = 632.8 nm) from Malvern (UK). Transmission electron microscopy (TEM) experiments were performed on a Philips CM100 TEM equipped with a TENGRA 2.3 K×2.3 K camera for digital imaging, and Philips Tecnai G2 20 S-TWIN with Gatan MultiScan model 794 for digital imaging.

Syntheses

5,10,15,20-Tetraphenylporphyrin,[S1] 4-formylbenzoic acid methyl ester,[S2] 5-(4-methoxycarbonylphenyl)-10,15,20-triphenylporphyrin,[S3] **Au1a**[S4] and complexes **3** and **4**[S5] were prepared according to the reported methods.

[Au(TPP–COO–PEG$_{5000}$–OCH$_3$)]Cl (1)

Complex **4** (22 mg, 0.026 mmol), HOBt (2.4 mg, 0.018 mmol) and PEG$_{5000}$–OCH$_3$ (124 mg, 0.025 mmol) were dissolved in anhydrous dichloromethane. With stirring at 0 °C, DCC (5.1 mg, 0.025 mmol) in dichloromethane was added to the solution mixture, followed by the addition of triethylamine (7.2 μL, 0.052 mmol). The solution mixture was then stirred at 0 °C for 2 h, and allowed to warm to room temperature and reacted for 5 days. After evaporation of volatile solvent under reduced pressure, the crude product was dissolved in deionized water, and undissolved solid was filtered. The aqueous solution was extracted with dichloromethane twice, and the combined organic layers were washed with dilute $HCl_{(aq)}$ solution twice followed by saturated $NaCl_{(aq)}$ solution. The organic layer was then dried over anhydrous sodium sulfate, filtered, and evaporated under reduced pressure. The crude product was dissolved in minimum amount of dichloromethane, and precipitated as a red solid by dropwise addition of the dichloromethane solution to diethyl ether. The red solid obtained after precipitation for totally three times was further purified by column chromatography on neutral alumina, using dichloromethane and methanol (50:1, v/v) as eluent. The product was obtained as red solid. Yield = 115 mg (78%). ^{1}H NMR (400 MHz, CDCl$_3$, 298 K): δ = 3.36 (s, 3H, –CH$_2$CH$_2$OC***H***$_3$), 3.44-3.89 (br, –C***H***$_2$C***H***$_2$O–), 3.98 (t, J = 4.8 Hz, 2H, –COOCH$_2$C***H***$_2$O–), 4.67 (t, J = 4.8 Hz, 2H, –COOC***H***$_2$CH$_2$O–), 7.83-7.92 (m, 9H, phenyl ***H***), 8.22-8.24 (m, 6H, phenyl ***H***), 8.34 (d, J = 8.0 Hz, 2H, –C$_6$***H***$_4$COO–), 8.54 (d, J = 8.0 Hz, 2H, –C$_6$***H***$_4$COO–), 9.20, (d, J = 5.2 Hz, 2H, pyrrolic ***H***), 9.27-9.29 (m, 6H, pyrrolic ***H***). MALDI-TOF MS (Fig. 1a): M_n = 5897 g mol^{-1}, M_w = 5971 g mol^{-1}, PDI = 1.01.

[Au(TPP–CONH–PEG$_{5000}$–OCH$_3$)]Cl (2)

The procedure was similar to that for complex **1**, except that H$_3$CO–PEG$_{5000}$–NH$_2$•HCl (124 mg, 0.025 mmol) was used instead of HO–PEG$_{5000}$–OCH$_3$. The red solid obtained after precipitation was further purified by column chromatography on neutral alumina, using dichloromethane and methanol (50:1, v/v) as eluent. The product was obtained as red solid. Yield = 88 mg (60%). ^{1}H NMR (400 MHz, CDCl$_3$, 298 K): δ = 3.38 (s, 3H, –CH$_2$CH$_2$OC***H***$_3$), 3.50-3.82 (br, –C***H***$_2$C***H***$_2$O–), 3.88 (br, 4H, –CONHC***H***$_2$C***H***$_2$O–), 7.83-8.00 (m, 9H, phenyl ***H***), 8.19-8.28 (m, 6H, phenyl ***H***), 8.30 (d, J = 8.2 Hz, 2H, –C$_6$***H***$_4$CONH–), 8.39 (s, 1H, –CON***H***CH$_2$CH$_2$O–), 8.53, (d, J = 8.2 Hz, 2H, –C$_6$***H***$_4$CONH–), 9.24-9.38 (m, 8H, pyrrolic ***H***). MALDI-TOF MS (Fig. 1b): M_n = 6062 g mol^{-1}, M_w = 6106 g mol^{-1}, PDI = 1.01.

Preparation of nanostructures of 1 and 2. Complex **1** or **2** (2.5 mg) was dissolved in acetonitrile (0.1 mL). With magnetic stirring at 700 rpm, the complex solution was added to PBS solution (1.9 mL). The solution mixture was stirred at room temperature for 15 min, and the volatile solvent was removed under reduced pressure. Precipitates, if any, were removed by centrifugation at 200 rpm for 5 min, and the supernatant was dialyzed against PBS solution using semipermeable minidialysis tubes (molecular weight cut-off = 1,000 Da; GE Healthcare) for 2 days. The purified nanostructures in PBS solution were used immediately for biological assays and other experiments.

The gold content of the nanostructures (10 µL) was determined by dissolving the nanostructures in conc. HNO$_3$ / conc. HCl solution mixture (9:1, v/v; 30 µL), followed by ICP-MS analysis.

Preparation of nanocomposites of 1 and DOX. The procedure was similar to that of the preparation of nanostructures of **1**, except that both complex **1** (2.5 mg) and **DOX** were dissolved in the acetonitrile (0.5 mL).

The gold content of the nanocomposites was determined by dissolving the nanocomposites in conc. HNO_3 / conc. HCl solution mixture (9:1, v/v; 30 μL), followed by ICP-MS analysis.

The content of **DOX** in the nanocomposites was measured as follows. The nanocomposite (10 μL) was dissolved in acetonitrile (90 μL), and subjected to gentle shaking overnight. The solution mixture was then dried under vacuum, and re-dispersed in 0.1 vol% formic acid in water/acetonitrile (7/3, v/v; 1 mL) solution mixture. The solution was then diluted and filtered, and the content of **DOX** in the nanocomposite was determined by UPLC with photodiode array detector using daunorubicin hydrochloride (3.6 μM) as the internal standard. The mobile phase was 0.1 vol% formic acid in water/acetonitrile (7:3, v/v) solution mixture. Calibration was done by dividing the peak areas of different concentrations of doxorubicin hydrochloride standard over the peak area of daunorubicin hydrochloride (3.6 μM) in the UPLC chromatograms of the doxorubicin hydrochloride standard spiked with daunorubicin hydrochloride (3.6 μM). The encapsulation efficiency of **DOX** by nanostructures of **1** was calculated by the equation:

Encapsulation efficiency = (mass of **DOX** in the nanocomposite) / (mass of **DOX** added in the preparation of nanocomposite) × 100%

***In vitro* cytotoxicity assays.** HeLa, NCI-H460, HCT116, A2780, A2780cis and A2780adr (4×10^3 cells), CCD-19Lu (1.0×10^4 cells) and MIHA and NCM460 (1.2×10^4 cells), respectively, were seeded in 96-well plates for 24 h. The cells were then treated with a serial concentration of gold(III) complexes and incubated at 37 °C under a 5% CO_2 atmosphere for 72 h. For nanostructures of **1**, **2** and **NC1**, they were dissolved in culture medium-PBS solution mixture, while complexes **3** and **4**, **Au1a**, **DOX** and cisplatin were dissolved in culture medium-DMSO solution mixture (99:1, v/v).

After incubation for 72 h, MTT in PBS solution (5 mg/mL, 10 μL) was added to each well. The plates were incubated at 37 °C under a 5% CO_2 atmosphere for another 4 h, and the cells were lysed by the addition of SDS solution (10%, w/w) with 0.01 M HCl (100 μL). After incubation in dark at 37 °C overnight, the cell viability after treatment was determined by a microtitre plate reader monitoring at the absorbance at 590 nm,

which reflected the amount of blue formazan formed. IC_{50} values were then determined by CompuSyn[S6] from three independent measurements.

Study on the release of hydrolyzed product from 1 and 2 in aqueous buffer solutions by ICP-MS. The nanostructures of gold(III) complex was dissolved in PBS solution (pH 7.4; 1 mg/mL) or sodium acetate buffer solution (pH 4.0; 1 mg/mL) and was dialyzed against 1 L of the buffer solution at 37 ^{o}C using semipermeable minidialysis tubes (molecular weight cut-off = 1,000 Da; GE Healthcare). At a predetermined time, an aliquot of solution was removed and dissolved in HNO_3/HCl solution mixture (9:1, v/v; volume of aliquot: volume of acid mixture = 1:3). The solution mixture was heated at 65 ^{o}C with shaking overnight, and the gold content was determined by ICP-MS from three independent aliquots.

Study on the hydrolysis of 1 and 2 in aqueous buffer solutions by ^{1}H NMR. The gold complex was dissolved in pH 4 or pH 7.4 buffer solution, and was incubated at 37 ^{o}C for 24 h. The solution was then dried under vacuum, and the solid was dissolved in $CDCl_3$ for ^{1}H NMR experiments.

Analysis of cell lysate of HCT116 cells treated with complexes 1 and 3 by UPLC-QTOF-MS. HCT116 cells (2×10^5 cells) were grown on 6-well plates at 37 ^{o}C under an atmosphere of 5% CO_2 for 48 h. The culture medium was then removed, washed with PBS solution, and incubated with culture medium containing nanostructures of **1** (2 μM), **3** (2 μM; with 1 vol% DMSO) or the solvent control for 24 h. All the cells were harvested, washed with PBS solution for three times and dispersed in ice-cold water. The cell suspension was then added to ice-cold acetonitrile (1:3, v/v), vortexed for 5 min, and centrifuged at 10,000 rpm at 4 ^{o}C. The supernatant was collected, dried under vacuum and the solid was re-dissolved in acetonitrile (HPLC grade) for UPLC-QTOF-MS experiments. The mobile phase was a mixture of 0.1% formic acid in water (phase A) and 0.1% formic acid in acetonitrile (phase B). Separation was achieved by using: 60% phase B from 0 to 2 min; 60–80% phase B from 2 to 15 min; 80% phase B from 15 to 20 min; 80–100% phase B from 20 to 20.1 min; 100% phase

B from 20.1 to 25 min and returned to initial conditions and equilibrated for 3 minutes. The flow rate was 0.4 mL/min and the injection volume was 2 μL. The UPLC-QTOF-MS chromatograms of **1**, **3**, **4** and solvent control in acetonitrile were also recorded for the interpretation of UPLC-QTOF-MS chromatograms of the cell lysate.

Cellular uptake experiments. HCT116, A2780 and A2780adr (2×10^5 cells), and MIHA and NCM460 (3.5×10^5 cells) cells were grown on 6-well plates at 37 ^oC under an atmosphere of 5% CO_2 for 48 h. The culture medium was then removed, washed with PBS solution, and incubated with culture medium containing nanostructures of **1** (2 μM), **NC1** ([**1**] = 2 μM; [**DOX**] = 0.29 μM), **3** (2 μM; with 1 vol% DMSO) and **Au1a** (2 μM; with 1 vol% DMSO). At a predetermined time, the culture medium was removed and washed with PBS solution for three times. The cells were then harvested and dispersed in ice-cold water. An aliquot of the cell suspension was subjected to Bio-rad protein assay with a microtitre plate reader at the absorbance at 595 nm, using bovine serum albumin as the protein standard. Another aliquot of the cell suspension was dissolved in HNO_3/HCl solution mixture (9:1, v/v; volume of aliquot: volume of acid mixture = 1:3). The solution mixture was heated at 65 ^oC with shaking overnight, and the gold content was determined by ICP-MS. Three independent experiments were carried out at every time point.

Time-dependent assays of induction of apoptosis of HCT116 and NCM460 cells by nanostructures of 1 and Au1a. HCT116 (1×10^5 cells) and NCM460 (1.5×10^5 cells) were grown on 35 mm glass bottom dishes respectively. The cells were incubated at 37 ^oC under an atmosphere of 5% CO_2 for 48 h. The culture medium was then removed and washed with PBS solution. The cells were then incubated with culture medium containing nanostructures of **1** (2 μM) and CellEvent Caspase-3/7 Green ReadyProbes Reagent (30 μL), or **Au1a** (2 μM; with 1 vol% DMSO) and CellEvent Caspase-3/7 Green ReadyProbes Reagent (30 μL) at 37 ^oC under an atmosphere of 5% CO_2. Fluorescence images at different time intervals were recorded by the fluorescence microscope without washing the cells.

Fluorescence microscopy imaging of HCT116 and NCM460 cells after incubation with gold(III) complexes and FITC-annexin V staining. HCT116 (1×10^5 cells) and NCM460 (1.5×10^5 cells) were grown on 35 mm glass bottom dishes respectively. The cells were incubated at 37 °C under an atmosphere of 5% CO_2 for 48 h. The culture medium was then removed and washed with PBS solution. The cells were then incubated with culture medium containing nanostructures of **1** (2 μM) or **Au1a** (2 μM; with 1 vol% DMSO) at 37 °C under an atmosphere of 5% CO_2 for 24 h. The culture medium was then removed, and the cells were washed with PBS solution and annexin-binding buffer solution. The cells were then incubated with FITC-annexin V (10 μL) and propidium iodide (1.5 μM) in annexin-binding buffer solution (115 μL) at room temperature in dark for 15 min. The solution was then removed, and the cells were washed with annexin-binding buffer solution. Fluorescence images were then recorded at λ_{ex} = 470±20 nm and λ_{em} > 515 nm, and λ_{ex} = 546±6 nm and λ_{em} > 590 nm.

Analysis of induction of apoptosis in HCT116 and NCM460 cells after incubation with gold(III) complexes and FITC-annexin V staining by flow cytometry. HCT116 (3.0×10^5 cells) and NCM460 (4.0×10^5 cells) were grown on 60 mm culture dishes respectively. The cells were incubated at 37 °C under an atmosphere of 5% CO_2 for 48 h. The culture medium was then removed, washed with PBS solution. The cells were then incubated with culture medium containing nanostructures of **1** (2 μM) or **Au1a** (2 μM; with 1 vol% DMSO) at 37 °C under an atmosphere of 5% CO_2 for 24 h. All the cells were harvested, and washed with PBS solution and annexin-binding buffer solution. The cells were collected by centrifugation at 2000 rpm for 5 min, and re-dispersed in annexin-binding buffer solution with cell concentration of *ca.* 1×10^6 cells/mL. FITC- Annexin V (5 μL) was added to an aliquot of the cell suspension (100 μL), and the cells were incubated at room temperature in dark for 15 min. The solution mixture was then diluted by annexin-binding buffer solution (400 μL), kept in ice and analyzed by flow cytometry immediately.

Analysis of induction of apoptosis in HCT116 and NCM460 cells after incubation with gold(III) complexes by CellEvent Caspase-3/7 Green Detection Reagent staining and flow cytometry. The procedure is similar to that using FITC-Annexin V

staining, except that the harvested cells were re-dispersed in PBS solution with 2% BSA (w/w; 100 μL) and incubated with CellEvent Caspase-3/7 Green Detection Reagent (2.5 μM) at 37 ^{o}C for 30 min, and subsequently stained with propidium iodide (1.5 μM) at room temperature for 10 min.

Experiments on co-culture cell model of HCT116 and NCM460 cells. HCT116 and NCM460 cells were grown on 100 mm culture dishes at 37 ^{o}C under an atmosphere of 5% CO_2. After reaching 80% confluency, the culture medium was removed. The NCM460 cells were washed with PBS solution twice and incubated with CMF_2HC dye (15 μM) in serum-free RPMI-1640 at 37 ^{o}C under an atmosphere of 5% CO_2 for 45 min. The solution was then removed. The stained NCM460 cells were washed with PBS solution for three times, trypsinized and seeded in 60 mm culture dishes (4.0×10^5 cells) or 35 mm glass bottom dishes (1.5×10^5 cells) with HCT116 cells (3.0×10^5 cells for 60 mm culture dishes; 1×10^5 cells for 35 mm glass bottom dishes). The cells were incubated at 37 ^{o}C under an atmosphere of 5% CO_2 for 48 h. The cells in 60 mm culture dishes and 35 mm glass bottom dishes were then incubated with culture medium containing nanostructures of **1** (2 μM) and subjected to flow cytometric analysis and fluorescence microscopy respectively, according to the procedure described above.

Another co-culture model with HCT116 cells pre-treated with CMF_2HC dye was prepared similarly, except that HCT116 cells were incubated with CMF_2HC dye (15 μM) in serum-free RPMI-1640 at 37 ^{o}C under an atmosphere of 5% CO_2 for 45 min, instead of NCM460 cells.

***In vivo* inhibition of tumor growth in nude mice bearing HCT116 xenograft.** The HCT116 xenograft nude mice model was constructed by injecting 1×10^6 cells to the right fore of BALB/c nude mice. When the tumor volume reached 70 mm^3 after inoculation, the tumor-bearing mice were used for animal study. The HCT116 xenograft nude mice were randomly divided into 4 groups (n = 8 per group). Different dosages of **1** in PBS or **3** in PBS/PET (60% polyethylene glycol 400, 30% ethanol, 10% Tween 80) mixture were injected to the nude mice by intravenous injection once

every 2 days for 24 days. The control group received equal volume of PBS only. During the treatment period, the tumor volume and body weight of the nude mice were measured per two days. The tumor volume was calculated by the equation: Volume (mm^3) = $l \times w^2/2$, where l and w were the length and width of the tumor. At the end of the treatment, the nude mice were sacrificed and the tumor weight was measured.

H&E staining and immunohistochemical (IHC) analysis of mice tissues. For histological and immunohistochemical analysis, the tissues were fixed with 3.7% formaldehyde, embedded with paraffin and sectioned. Subsequently, the tissues were stained with hematoxylin and eosin for histological observation under an optical microscope (magnification: 10×). The expression of p53, Ki67, VEGFR2 and TUNEL was determined by IHC methods as reported previously.[S7]

Hematological analysis of nude mice bearing HCT116 xenograft. The blood samples of mice without bearing HCT116 xenograft and xenograft-bearing mice treated with **1**, **3** and PBS control were collected. The plasma was obtained by centrifuging the blood sample with speed at 3000 rpm. The plasma was then subjected to hematological analysis to determine biochemical indexes of alanine aminotransferase (ALT), asparatate aminotransferase (AST), globulin (GLB), blood urea nitrogen (BUN), uric acid (UA), creatinine (CREA), lactate dehydrogenase (LDH) and creatine kinase (CK) in blood of the mice.

***In vivo* inhibition of tumor growth in nude mice bearing A2780cis xenograft.** The A2780cis xenograft nude mice model was constructed by injecting 2.5×10^6 cells to the right back flank of female BALB/c nude mice. When the tumor volume reached around 40 mm^3 after inoculation, the tumor-bearing mice were used for animal study. The A2780cis xenograft nude mice were randomly divided into 2 groups (n = 5 per group). **1** in PBS (4 mg/kg) or PBS solvent control were injected to the nude mice by intravenous injection once every 2–3 days for 13 days. During the treatment period, the tumor volume and body weight of the nude mice were measured

once every 2–3 days. The tumor volume was calculated by the equation: Volume (mm^3) = $l \times w^2/2$, where l and w were the length and width of the tumor. At the end of the treatment, the nude mice were sacrificed and the tumor weight was measured.

Cellular uptake experiments of NC1 and DOX by A2780adr. A2780adr cells (2×10^5 cells) were grown on 6-well plates at 37 °C under an atmosphere of 5% CO_2 for 48 h. The culture medium was then removed, washed with PBS solution, and incubated with culture medium containing **NC1** ([**1**] = 3.5 μM and [**DOX**] = 0.5 μM) and **DOX** (0.5 μM; with 1 vol% DMSO) respectively. At a predetermined time, the culture medium was removed and washed with PBS solution for three times. The cells were then harvested and dispersed in ice-cold water. An aliquot of the cell suspension was subjected to Bio-rad protein assay with a microtitre plate reader at the absorbance at 595 nm, using bovine serum albumin as the protein standard. Another aliquot of the cell suspension was dissolved in ice-cold acetonitrile/water solution mixture (3:1, v/v), vortexed for 5 min, and centrifuged at 10,000 rpm at 4 °C. The supernatant was collected, dried under vacuum and the solid was re-dissolved in acetonitrile (HPLC grade) for UPLC-QTOF-MS experiments with daunorubicin hydrochloride (0.9 μM) as the internal standard. The mobile phase was 0.1% formic acid in water-acetonitrile solution mixture (4:1, v/v). Calibration was done by dividing the peak areas of different concentrations of doxorubicin hydrochloride standard over the peak area of daunorubicin hydrochloride (0.9 μM) in the chromatograms of the doxorubicin hydrochloride standard spiked with daunorubicin hydrochloride (0.9 μM).

Study on induction of apoptosis of HCT116 cells by NC1 and DOX. The induction of apoptosis of HCT116 cells was investigated by CellEvent Caspase-3/7 Green ReadyProbes Reagent (30 μL), similar to the procedure of the experiments on gold(III) complex **1**, except using **NC1** ([**1**] = 3.5 μM and [**DOX**] = 0.5 μM) or **DOX** (0.5 μM; with 1 vol% DMSO) instead of **1**.

Determination of gold content in tissues of mice treated with complex 1, 3 and Au1a at the end point of tumor growth inhibition study by ICP-MS. At the end

point of tumor growth inhibition study, the mice treated with complex **1**, **3** and **Au1a**, respectively, were sacrificed and tissues including liver, spleen, lung, kidney and heart were harvested. The tissues were digested with 10% HCl in concentrated HNO_3 (0.1 g of tissue/1 mL). The solution mixture was heated at 60 ^oC for 15 h, and then diluted 50 times by ultra-pure water for ICP-MS analysis.

Determination of *in vivo* biodistribution of complex 1, 3 and Au1a by ICP-MS. Nude mice bearing HCT116 xenografts were treated with **1** (13 mg/kg), or equal molar amount of **Au1a** (2 mg/kg) or **3** (2 mg/kg) through intravenous injection. After 24 h of the injection, the mice were sacrificed and tissues from tumor, liver, spleen, lung, kidney and heart were harvested. The tissues were digested with 10% HCl in concentrated HNO_3 (0.1 g of tissue/1 mL). The solution mixture was heated at 60 ^oC for 15 h, and then diluted 50 times by ultra-pure water for ICP-MS analysis.

Analysis of tumor tissue from mice treated with complexes 1 by UPLC-QTOF-MS. At the end point of tumor growth inhibition study, the mice treated with solvent control or complex **1** were sacrificed and the tumor tissues were harvested, followed by homogenization in 50% ice-cold acetonitrile (0.1 g/300 μL). The homogenate was then added to acetonitrile (1:3, v/v), vortexed for 5 min, and centrifuged at 10,000 rpm at 4 ^oC. The supernatant was collected, dried under vacuum and the solid was re-dissolved in acetonitrile for UPLC-QTOF-MS experiments. The mobile phase was a mixture of 0.1% formic acid in water (phase A) and 0.1% formic acid in acetonitrile (phase B). Separation was achieved by using: 60% phase B from 0 to 2 min; 60–80% phase B from 2 to 15 min; 80% phase B from 15 to 20 min; 80–100% phase B from 20 to 20.1 min; 100% phase B from 20.1 to 25 min and returned to initial conditions and equilibrated for 3 minutes. The flow rate was 0.4 mL/min and the injection volume was 2 μL.

References

S1. A. D. Adler, F. R. Longo, J. D. Finarelli, J. Goldmacher, J. Assour and L. Korsakoff, *J. Org. Chem.*, 1967, **32**, 476.

S2. J. M. Landesberg, M. A. Slam and M. Mandel, *J. Org. Chem.*, 1981, **46**, 5025.

S3. J. P. C. Tomé, M. G. P. M. S. Neves, A. C. Tomé, J. A. S. Cavaleiro, A. F. Mendonça, I. N. Pegado, R. Duarte and M. L. Valdeira, *Bioorg. Med. Chem.*, 2005, **13**, 3878.

S4. E. B. Fleischer and A. Laszlo, *Inorg. Nucl. Chem. Lett.*, 1969, **5**, 373.

S5. L. Sun, H. Chen, Z. Zhang, Q. Yang, H. Tong, A. Xu and C. Wang, *J. Inorg. Biochem.*, 2012, **108**, 47.

S6. T. C. Chou, *Pharmacol. Rev.*, 2006, **58**, 621.

S7. Y. Huang, L. He, W. Liu, C. Fan, W. Zheng, W. S. Wong and T. Chen, *Biomaterials*, 2013, **34**, 7106.

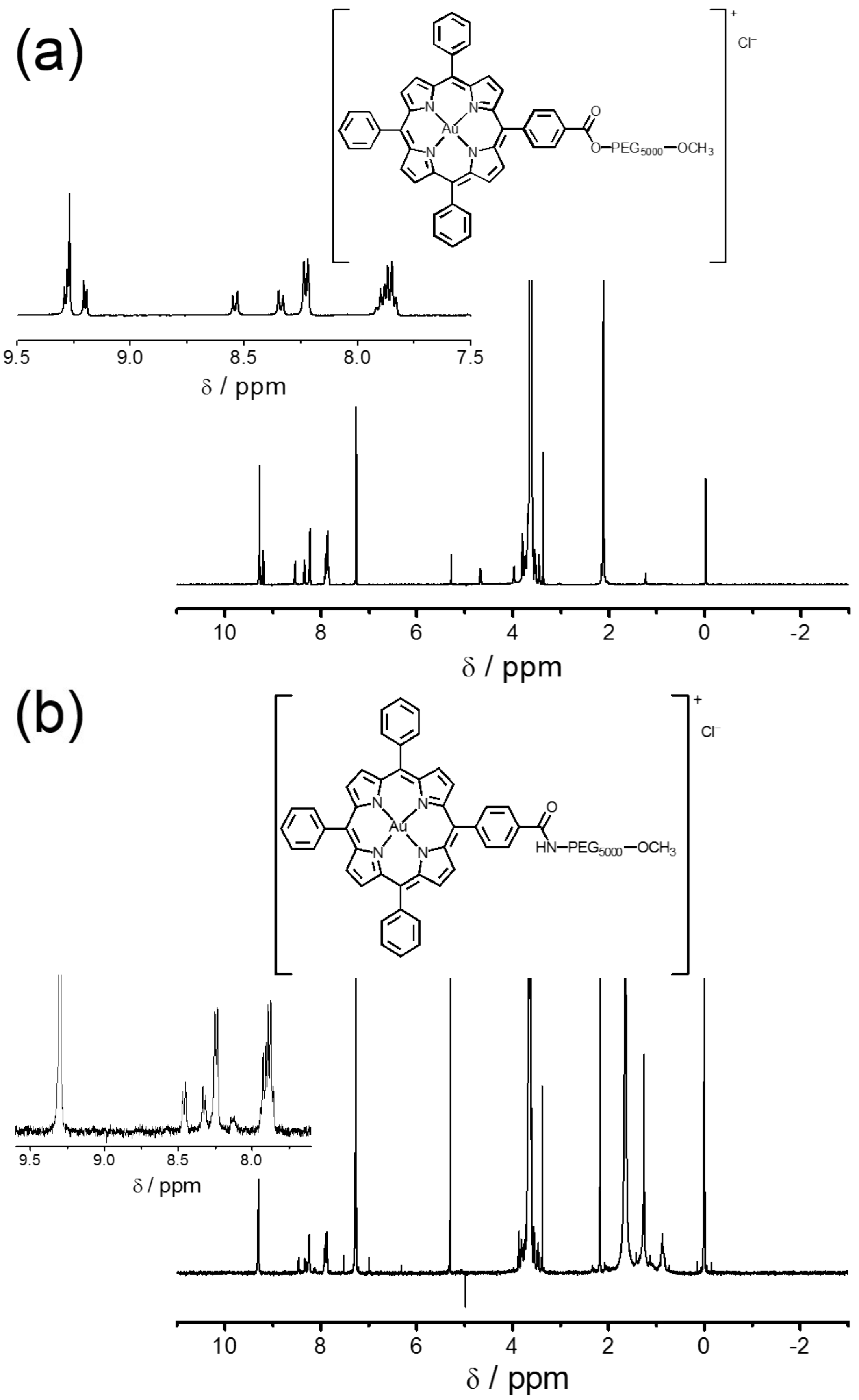

Fig. S1 ^{1}H NMR spectra of (a) **1** and (b) **2** in $CDCl_3$.

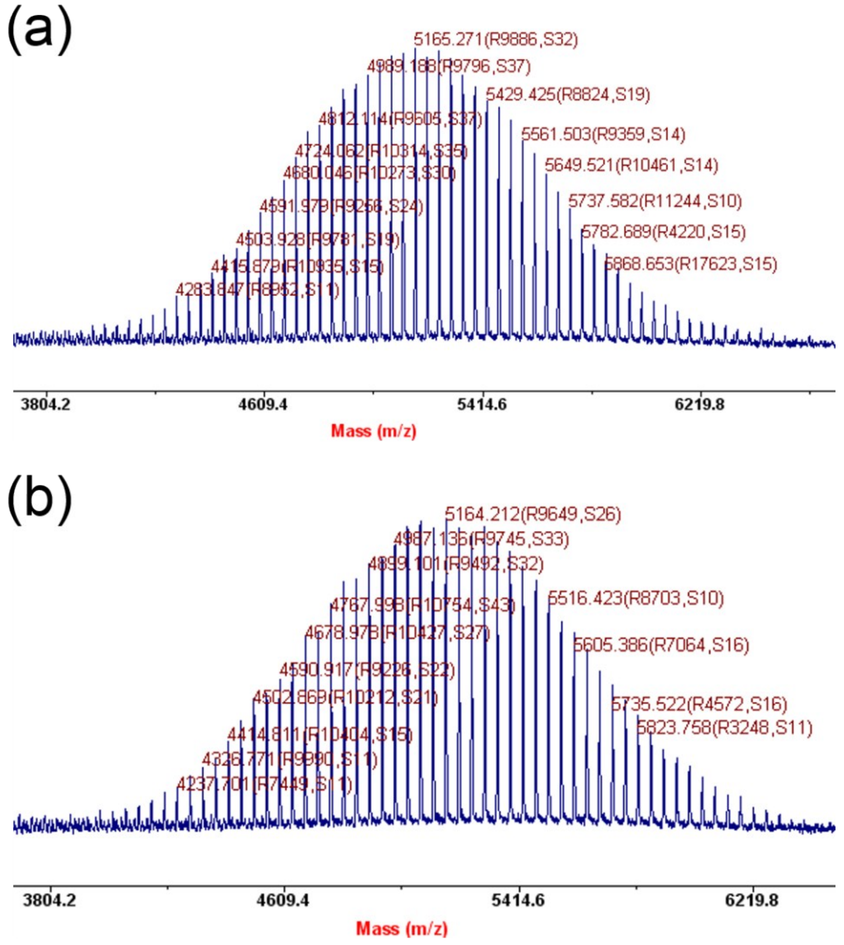

Fig. S2 MALDI-TOF MS of (a) H_3CO–PEG_{5000}–OCH_3 and (b) H_3CO–PEG_{5000}–NH_2•HCl, using α-cyano-4-hydroxycinnamic acid with sodium trifluoroacetate as the matrix.

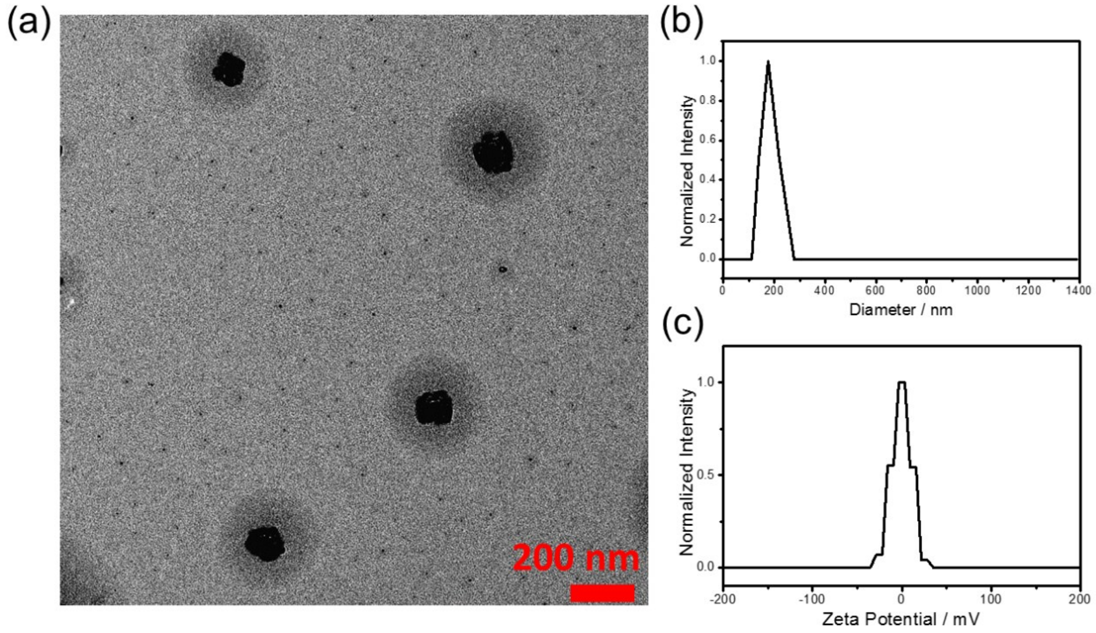

Fig. S3 (a) TEM image, (b) DLS profile and (c) profile showing the zeta potential of nanostructures of **2** in PBS solution.

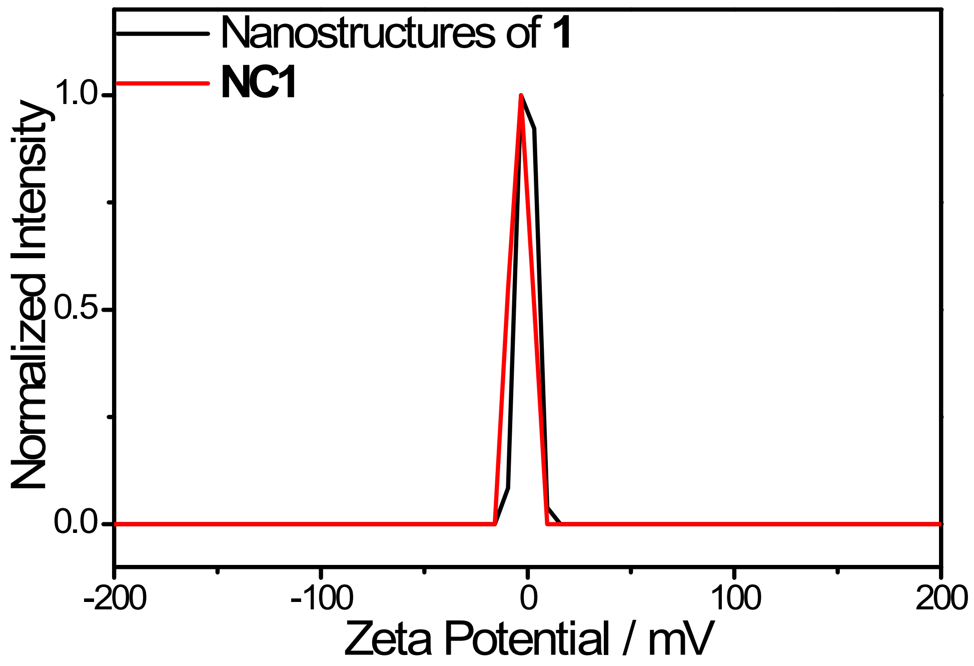

Fig. S4 Profiles showing the zeta potential of nanostructures of **1** and nanocomposite of **1** and **DOX** in PBS solution.

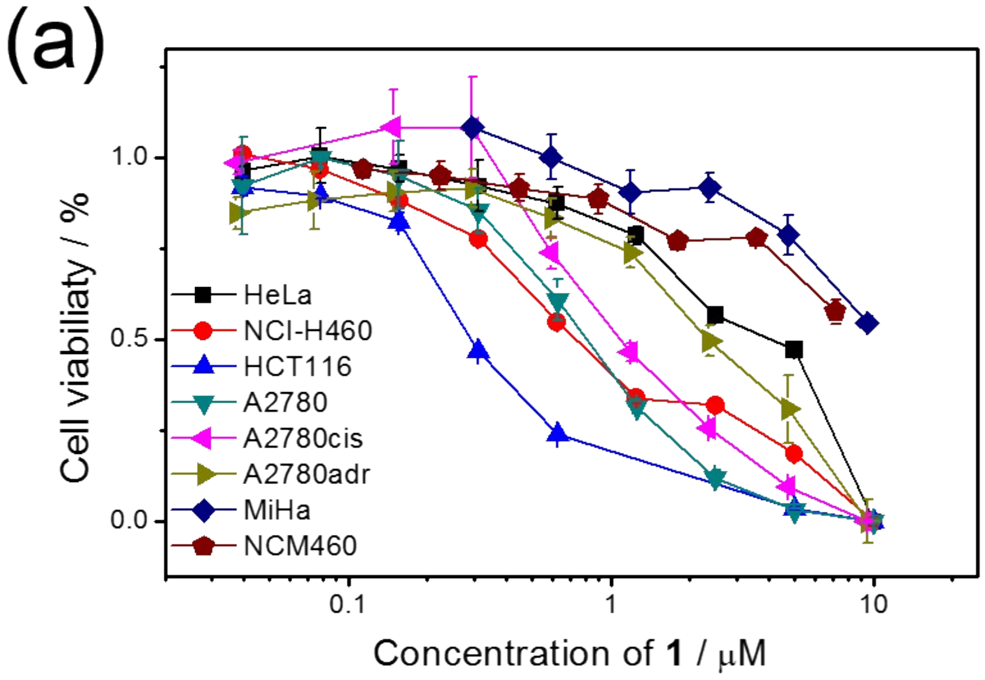

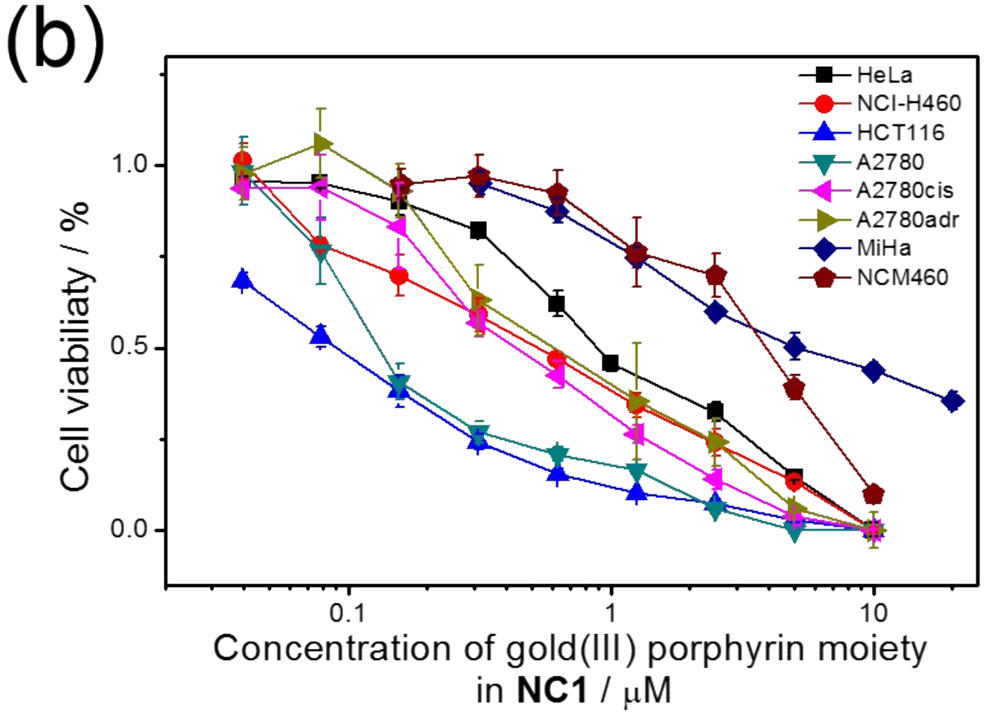

Fig. S5 Profiles showing cell viability upon treatment with different concentrations of (a) nanostructures of **1** and (b) nanocomposite (**NC1**) of **1** and **DOX** for 72 h.

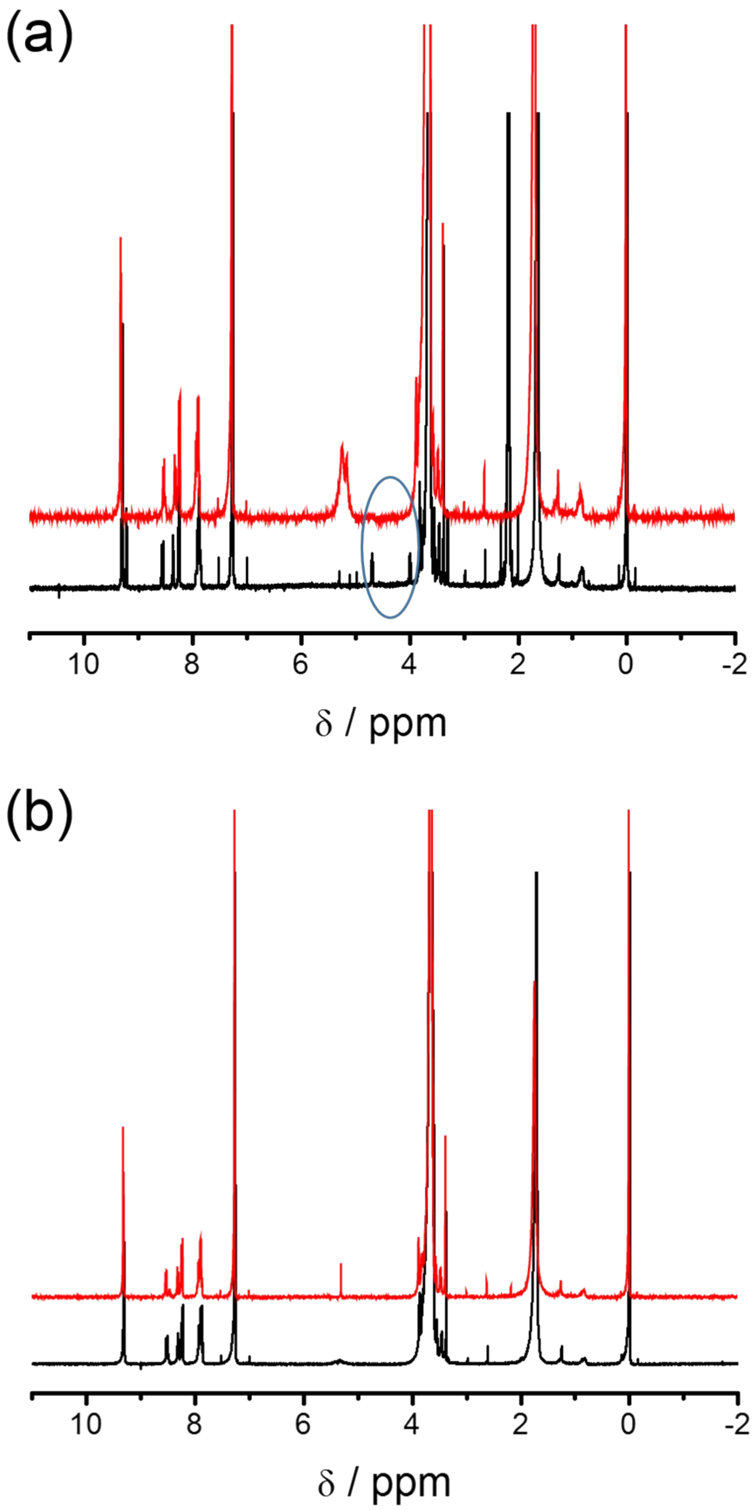

Fig. S6 (a) **1** and (b) **2** were incubated in (black) pH 7.4 and (red) pH 4 buffer solution, respectively, at 37 °C for 24 h. The solutions were then vacuum dried, and their ^{1}H NMR spectra were recorded in $CDCl_3$.

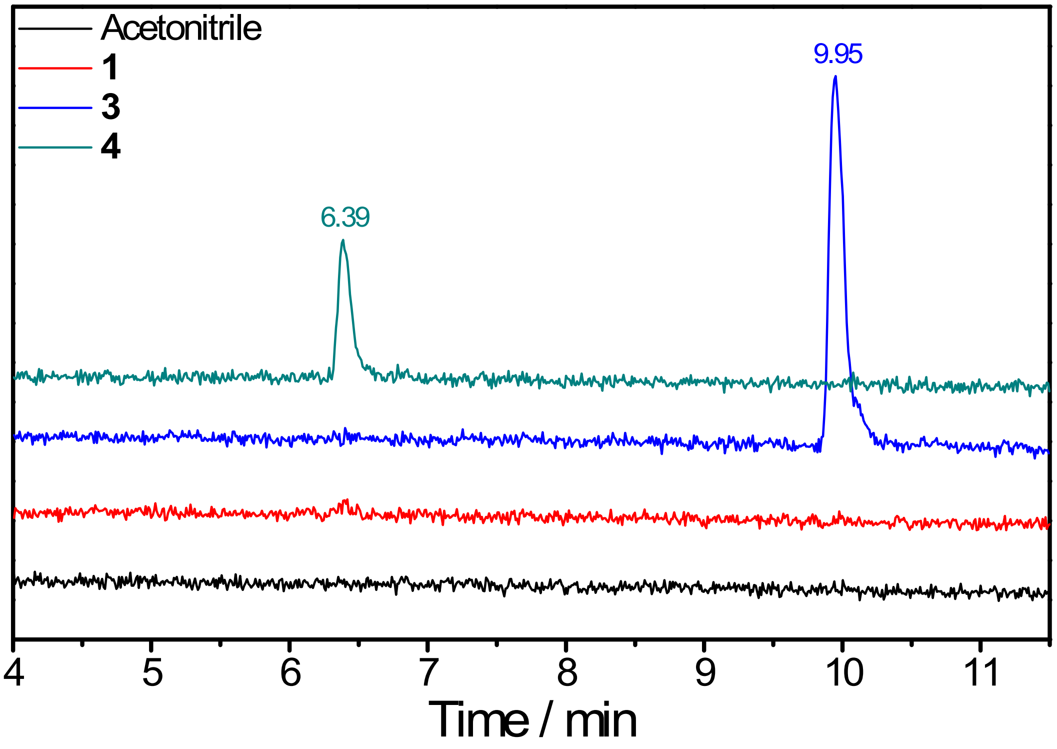

Fig. S7 Total-ion chromatograms of UPLC-QTOF-MS of **1**, **3** and **4** (1 μM) in acetonitrile solution.

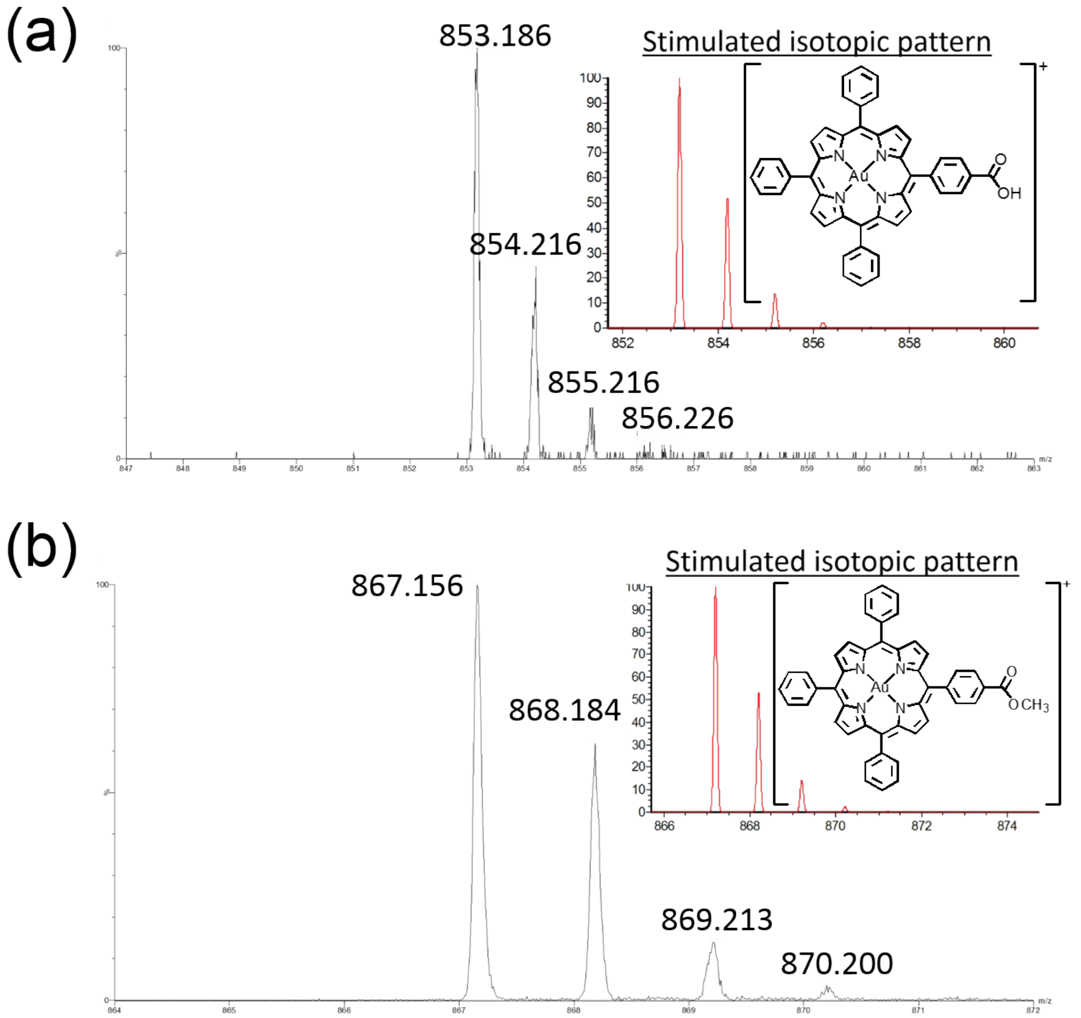

Fig. S8 MS recorded at t = (a) 6.42 min and (b) 9.94 min of the UPLC-QTOF-MS chromatogram of cell lysates of HCT116 cells treated with **3** (2 μM) for 24 h. Inset: Simulated isotopic pattern of (a) $[Au(TPP–COOH)]^+$ and (b) $[Au(TPP–COOCH_3)]^+$.

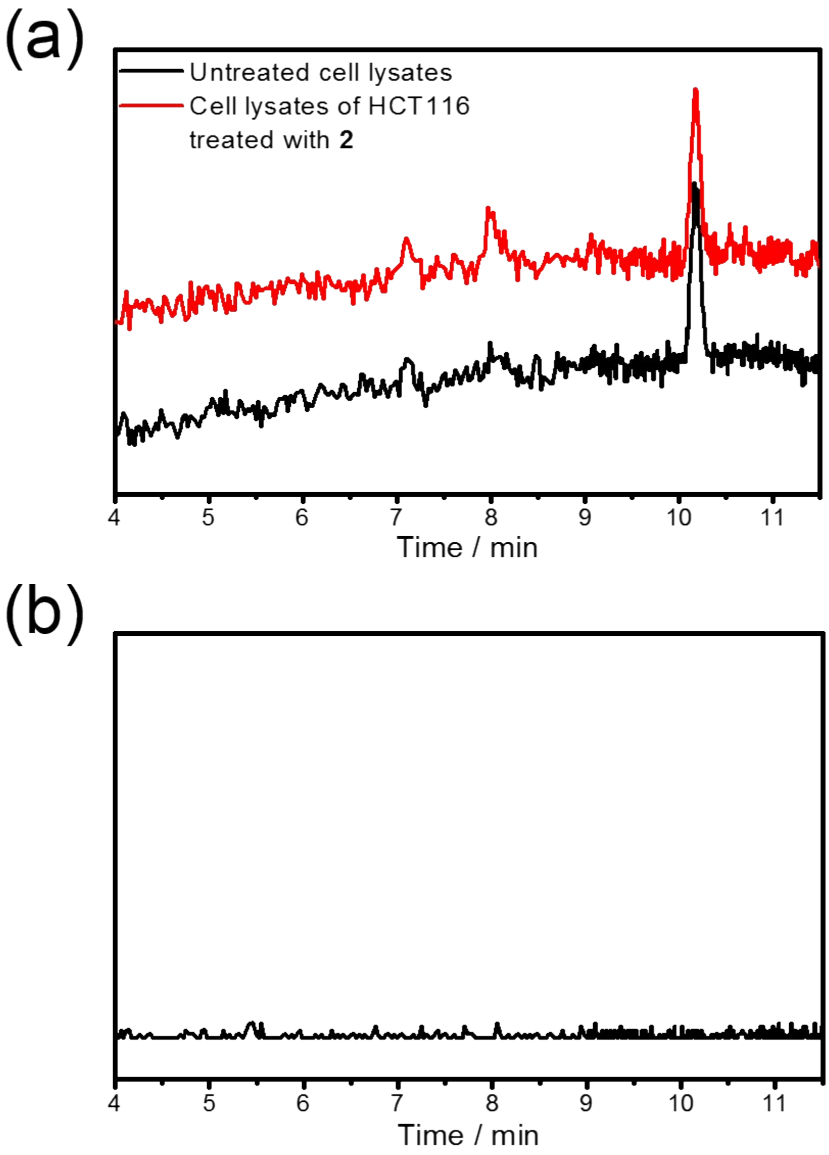

Fig. S9 (a) Total-ion chromatograms of UPLC-QTOF-MS of cell lysates of untreated HCT116 cells and HCT116 cells treated with **2** (2 μM), respectively, for 24 h. (b) Selected-ion chromatograms at *m/z* = 853, corresponding to the *m/z* of [Au(TPP–COOH)]$^+$.

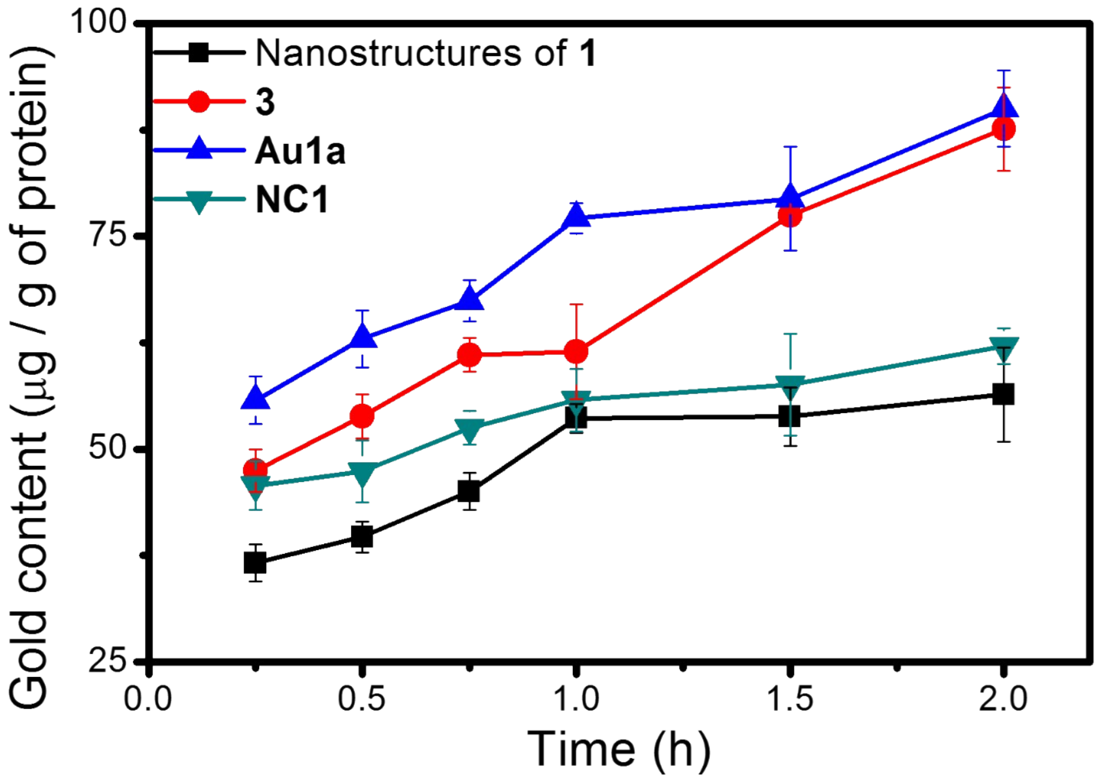

Fig. S10 Cellular uptake of the complexes by MIHA cells after incubation at 37 °C for indicated time intervals.

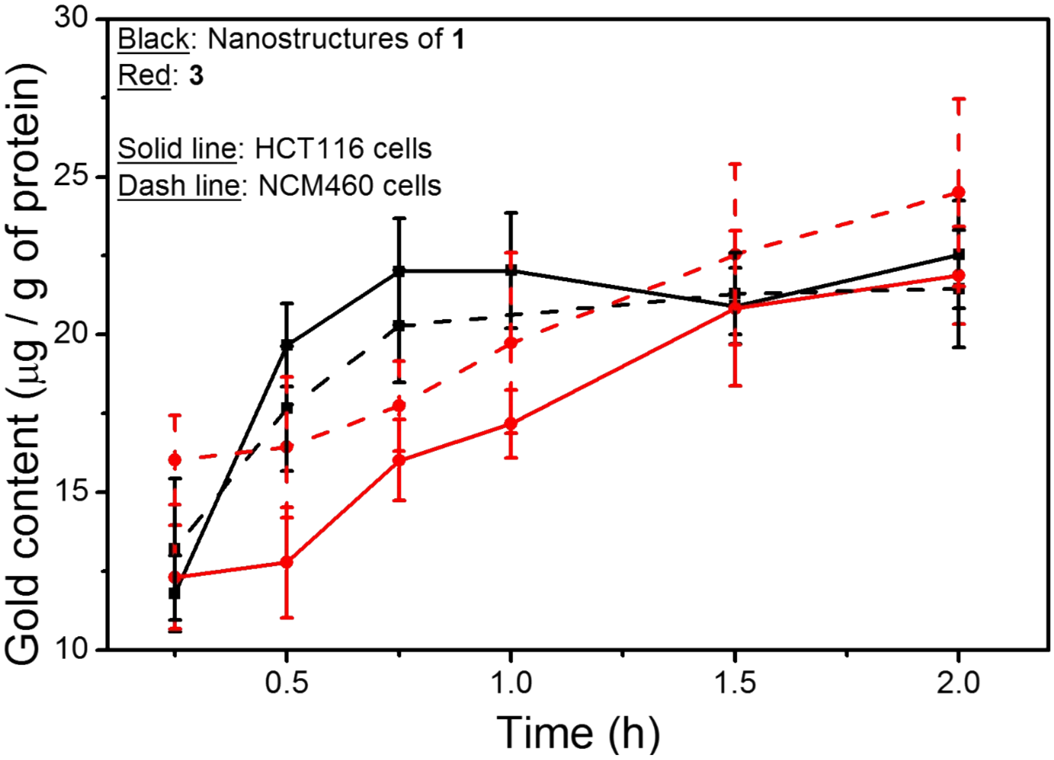

Fig. S11 Cellular uptake of **1** and **3** (2 μM) into HCT116 and NCM460 cells, respectively, after incubation at 4 °C for indicated time intervals.

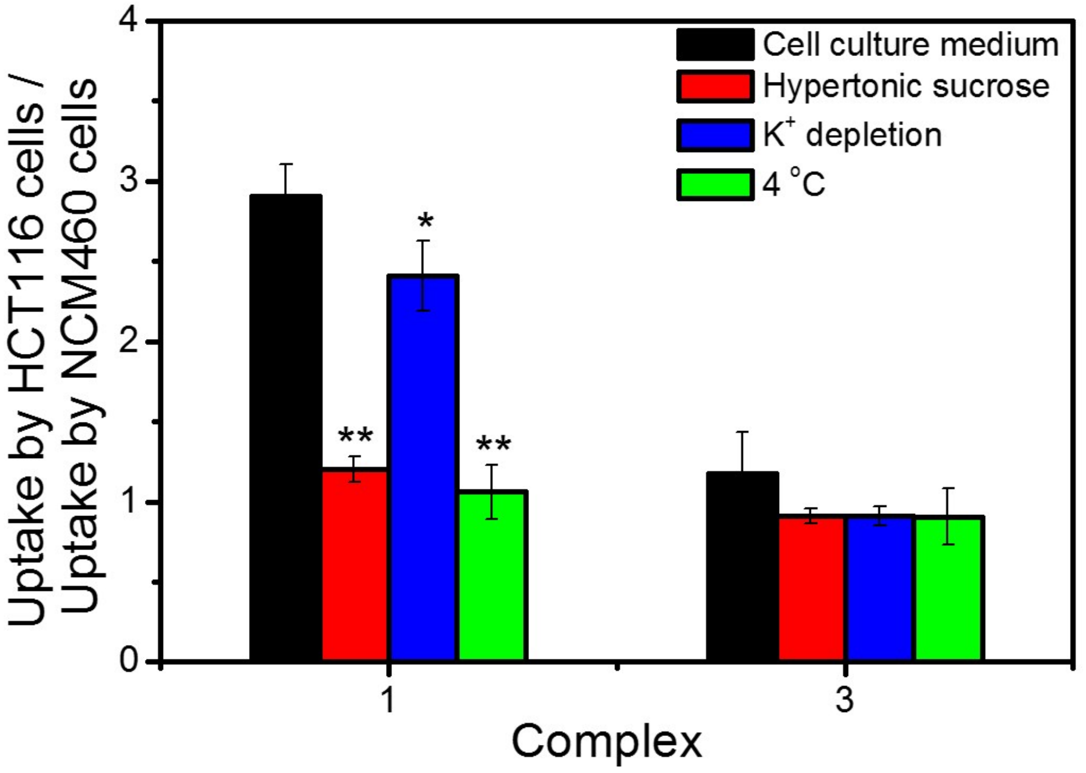

Fig. S12 Bar chart showing the ratio of cellular uptake of the complexes (2 μM) into HCT116 and NCM460 cells after incubation for 2 h in (black) cell culture medium with 10 vol% fetal bovine serum at 37 °C, (red) cell culture medium with 10 vol% fetal bovine serum and 0.45 M sucrose at 37 °C, (blue) aqueous buffer solution (140 mM NaCl, 20 mM HEPES, 1 mM $CaCl_2$, 1 mM $MgCl_2$ and 1 g/L D-glucose, pH 7.4) at 37 °C, and (green) cell culture medium with 10 vol% fetal bovine serum at 4 °C, respectively. * and ** denoted p <0.05 and < 0.01, respectively, vs. experiment with cell culture medium.

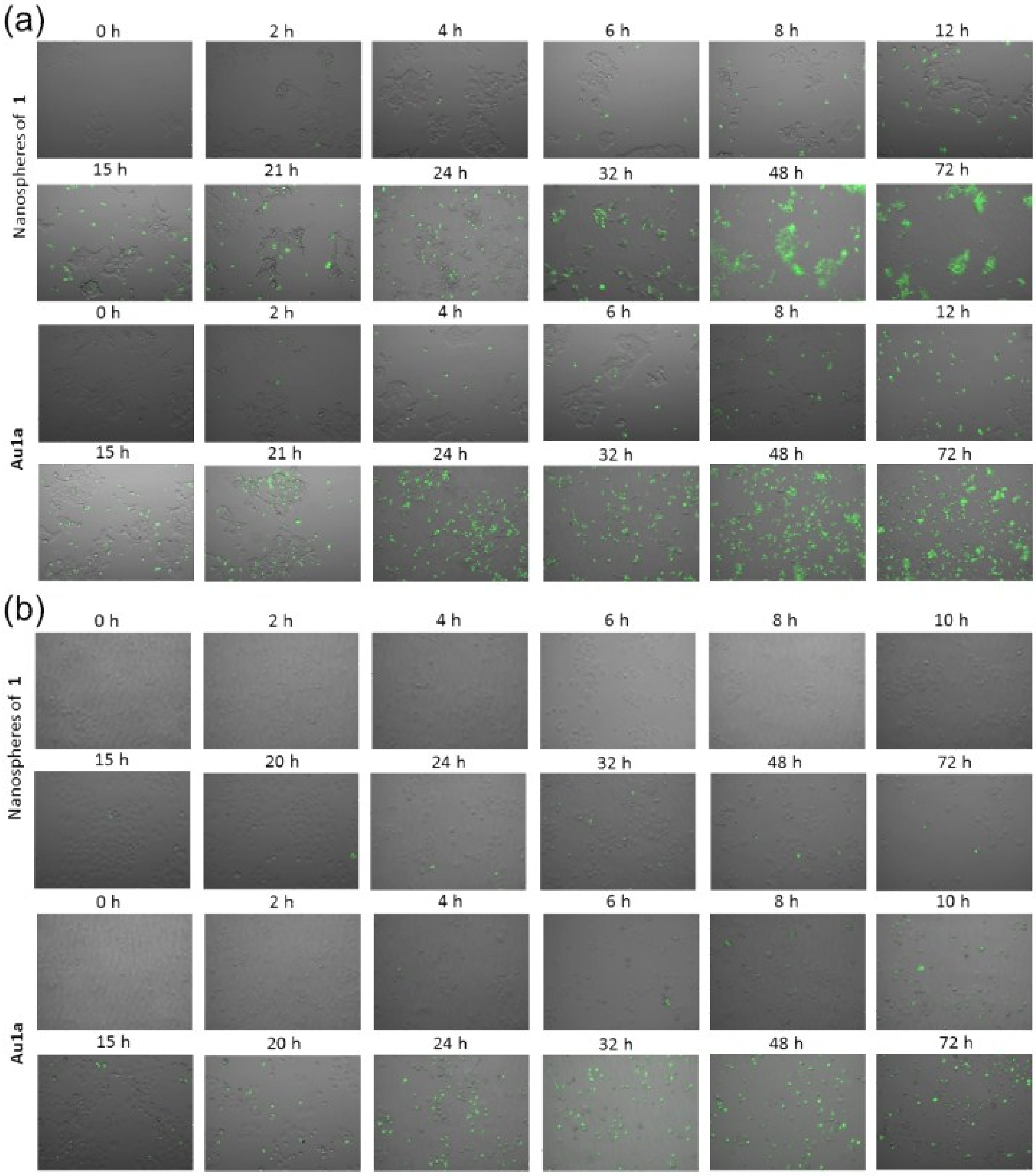

Fig. S13 Fluorescence microscopy images of (a) HCT116 cells and (b) NCM460 cells after incubation with CellEvent Caspase-3/7 Green ReadyProbes Reagent (30 µL), and nanostructures of **1** and **Au1a** (2 µM) respectively, at different time intervals, showing the induction of apoptosis of the two different cell lines by **1** and **Au1a**.

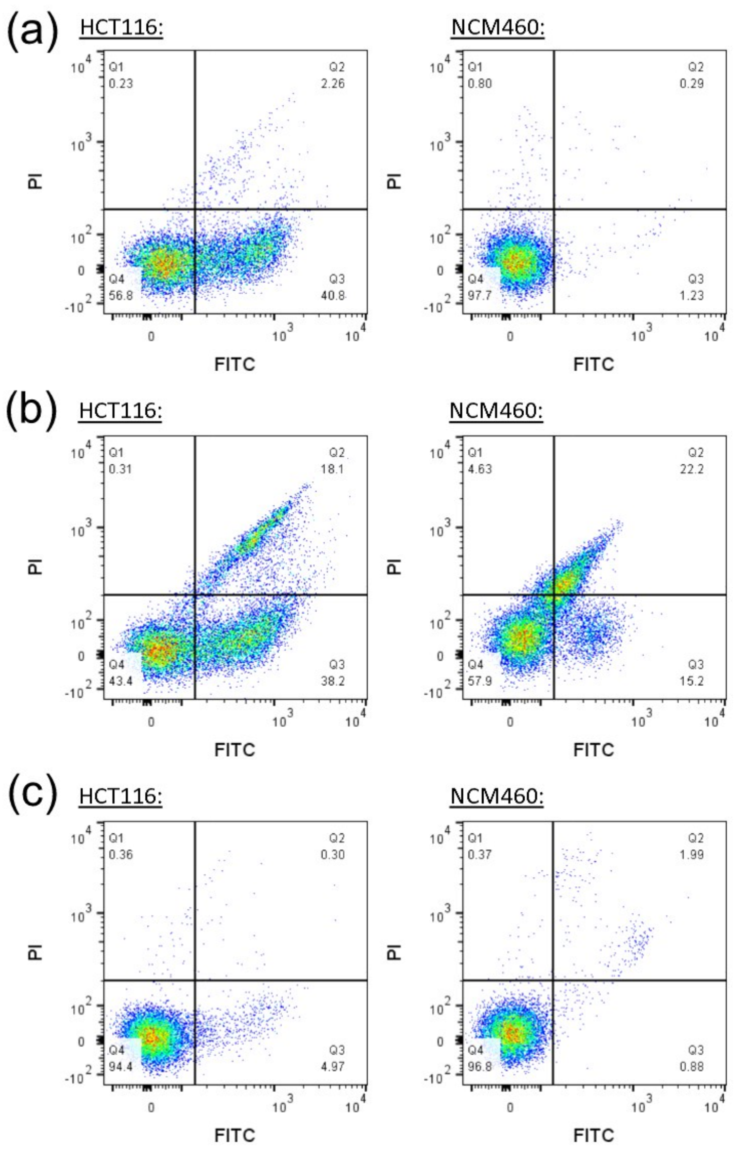

Fig. S14 Flow cytometric analysis of the induction of apoptosis of HCT116 and NCM460 cells based on CellEvent Caspase-3/7 Green Detection Reagent (2.5 μM) and propidium iodide (1.5 μM) staining. The HCT116 and NCM460 cells were incubated with (a) nanostructures of **1** (2 μM), (b) **Au1a** (2 μM) and (c) solvent control for 24 h.

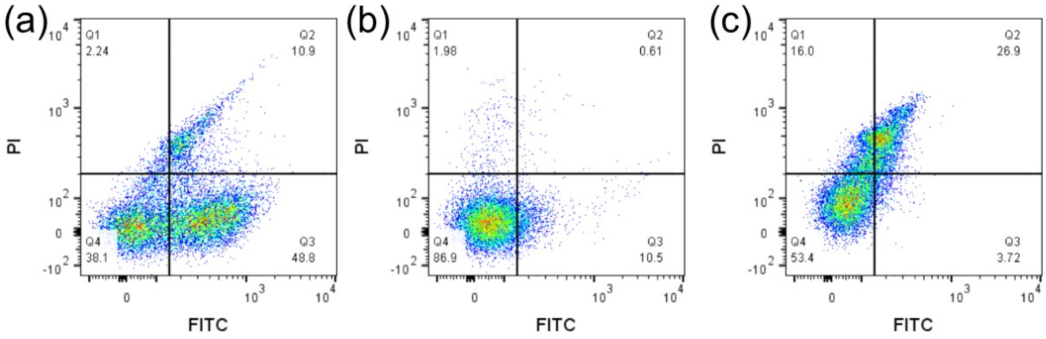

Fig. S15 (a) Flow cytometric analysis of HCT116 cells treated with nanostructures of **1** (2 μM) for 36 h after FITC-annexin V and propidium iodide staining. (b) Flow cytometric analysis of NCM460 cells treated with nanostructures of **1** (2 μM) for 36 h after FITC-annexin V and propidium iodide staining. (c) Flow cytometric analysis of NCM460 cells treated with **Au1a** (2 μM) for 36 h after FITC-annexin V and propidium iodide staining.

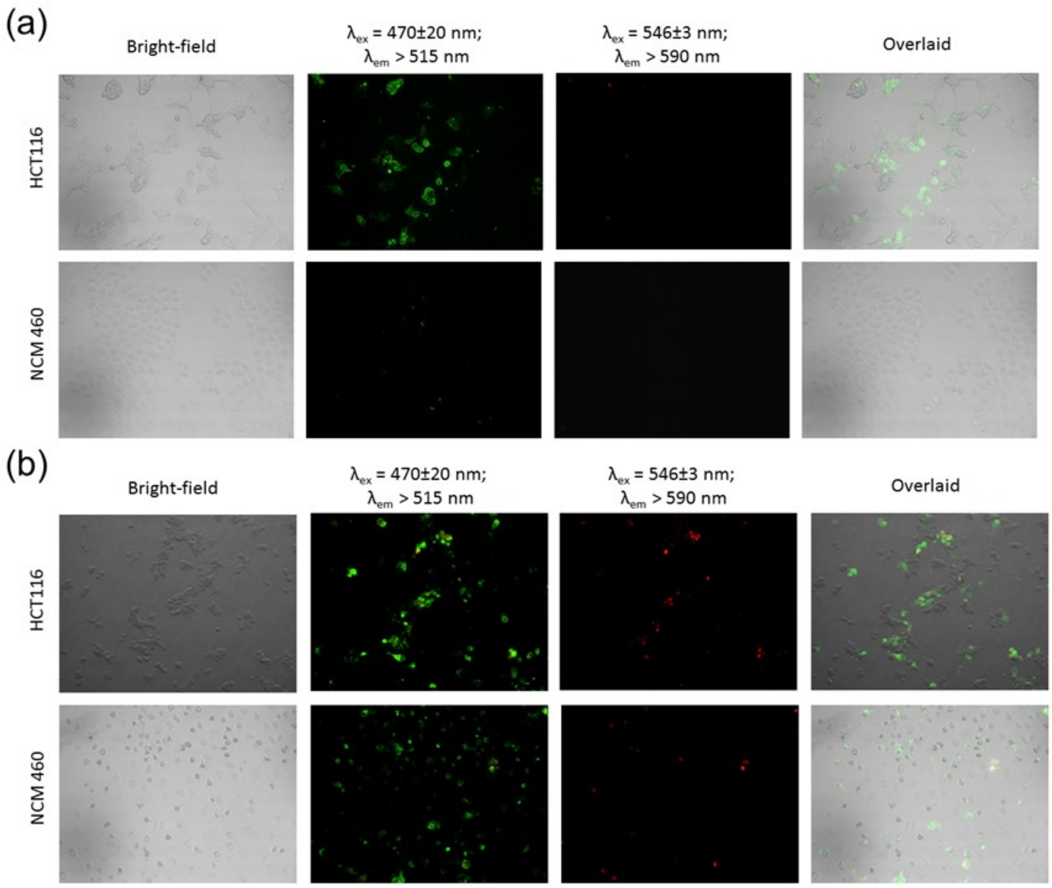

Fig. S16 Images of fluorescence microscopy of HCT116 and NCM460 cells stained with FITC-Annexin V and propidium iodide after incubation with complexes (a) **1** (2 μM) and (b) **Au1a** (2 μM), respectively, for 24 h. The overlaid images were prepared by ImageJ.

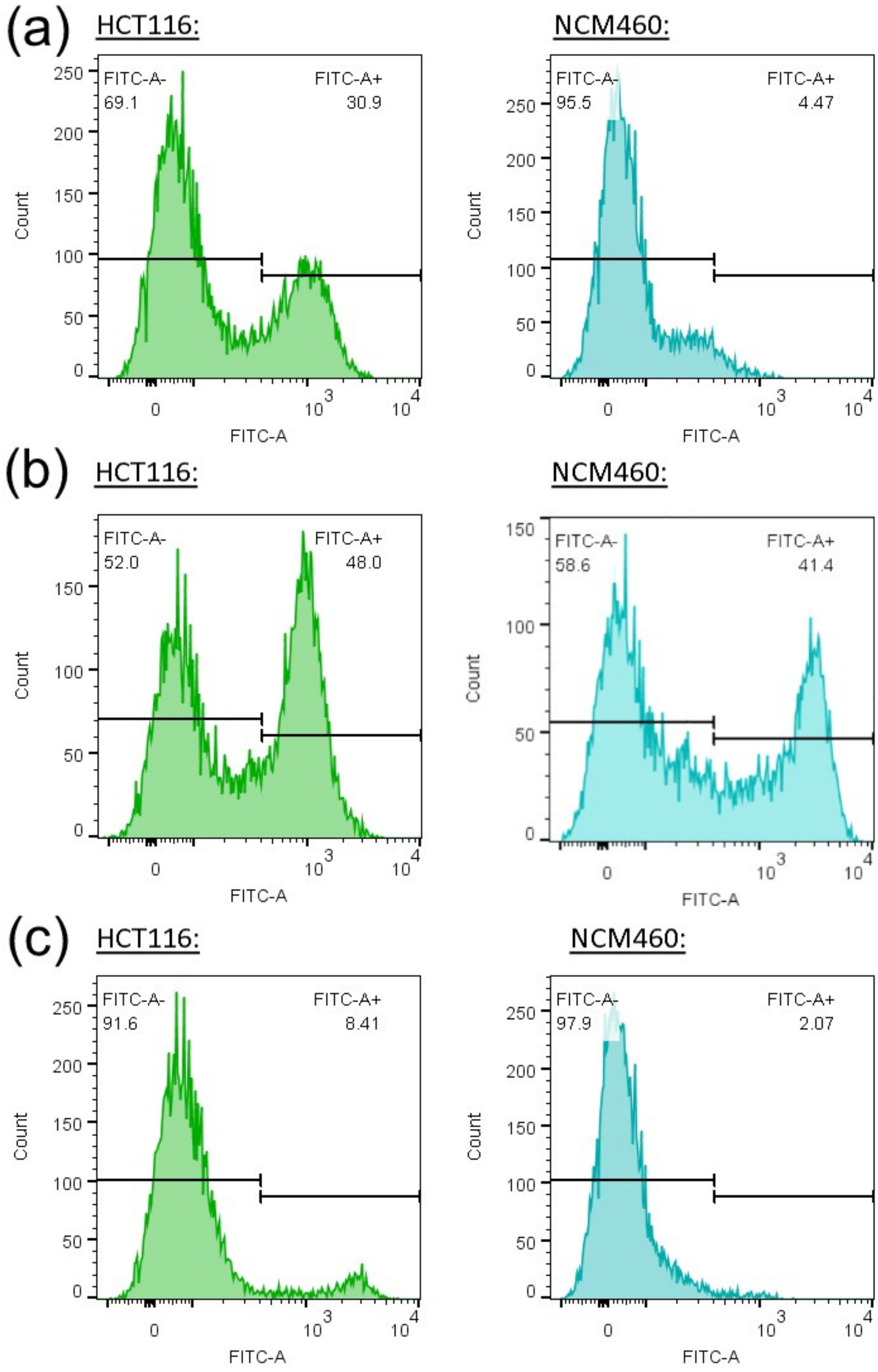

Fig. S17 Analysis of the induction of apoptosis of HCT116 and NCM460 cells by the gold complexes using FITC-Annexin V staining and flow cytometry. The HCT116 and NCM460 cells were incubated with (a) nanostructures of **1** (2 μM), (b) **Au1a** (2 μM) and (c) solvent control for 24 h.

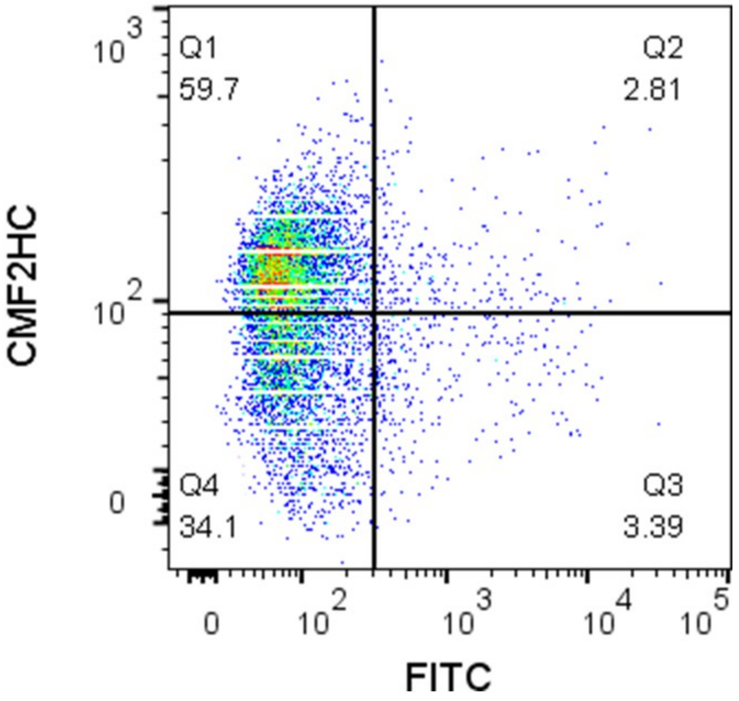

Fig. S18 Analysis by flow cytometry of the co-culture model of NCM460 cells, which were pre-treated by CMF_2HC dye, and HCT116 cells after incubation with solvent control for 24 h and subsequent FITC-Annexin V staining.

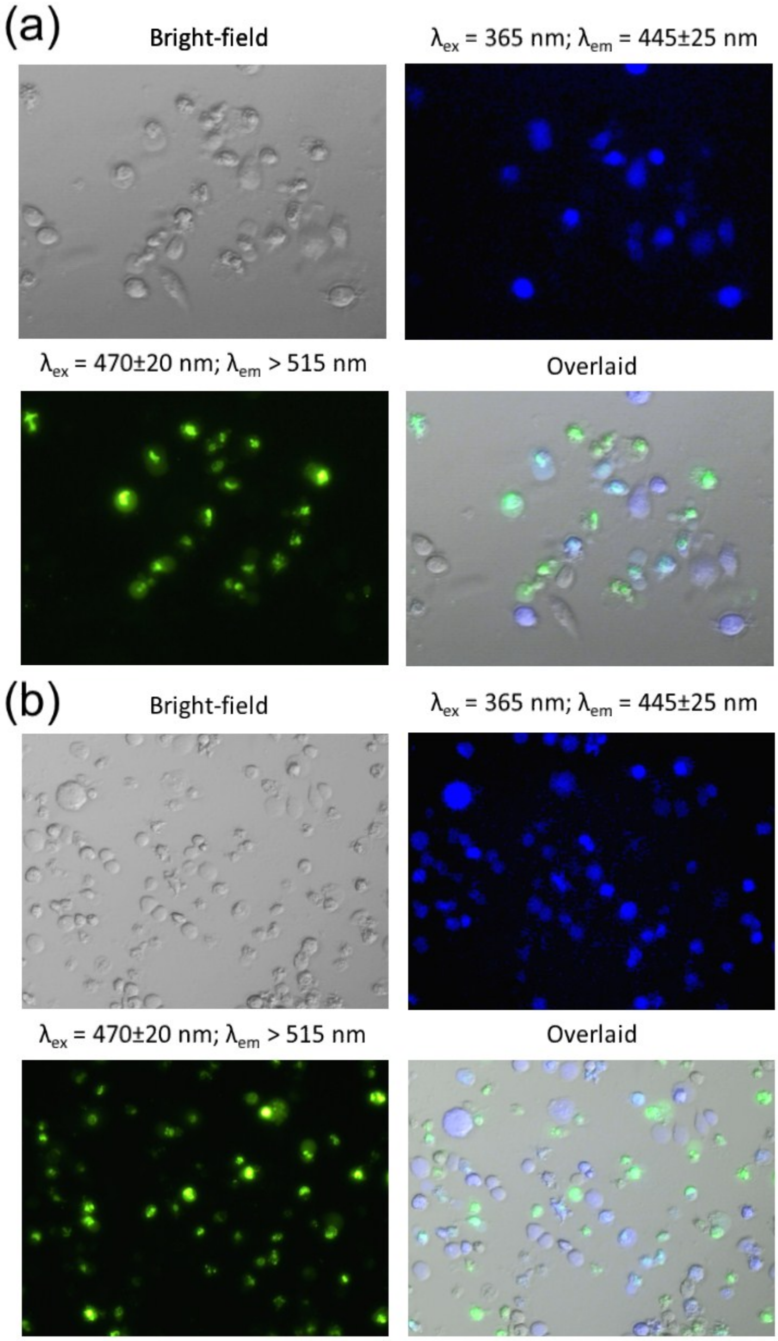

Fig. S19 Fluorescence microscopy images of co-culture model of HCT116 and NCM460 cells stained with CellEvent Caspase-3/7 Green ReadyProbes Reagent after incubation with **Au1a** (2 μM) for (a) 24 h and (b) 36 h respectively. The NCM460 cells were pre-treated with blue-emissive CMF_2HC dye for differentiation from HCT116 cells. The overlaid images were prepared by ImageJ.

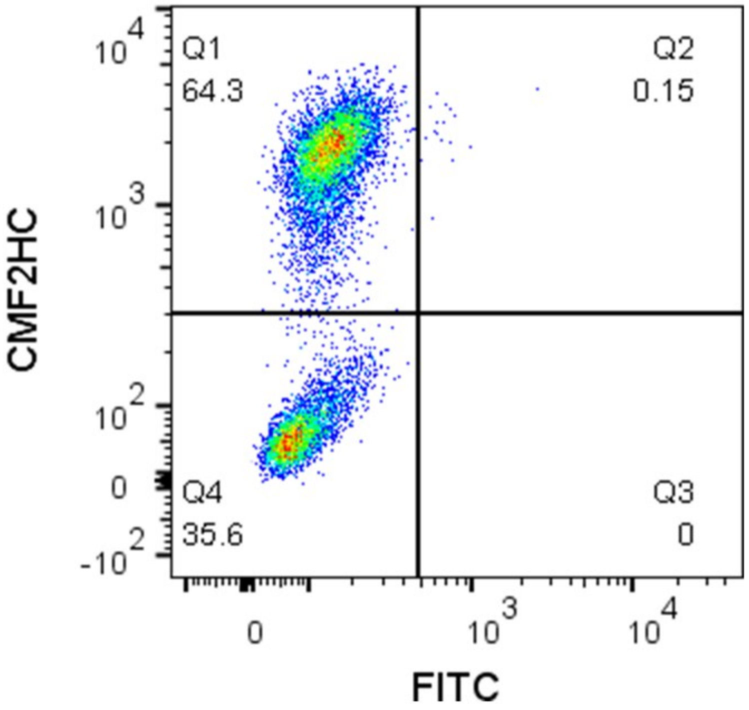

Fig. S20 Flow cytometric analysis of co-culture model of HCT116 and NCM460 cells treated with solvent control for 24 h. The HCT116 cells were pre-treated with blue-emissive CMF_2HC dye for differentiation from NCM460 cells.

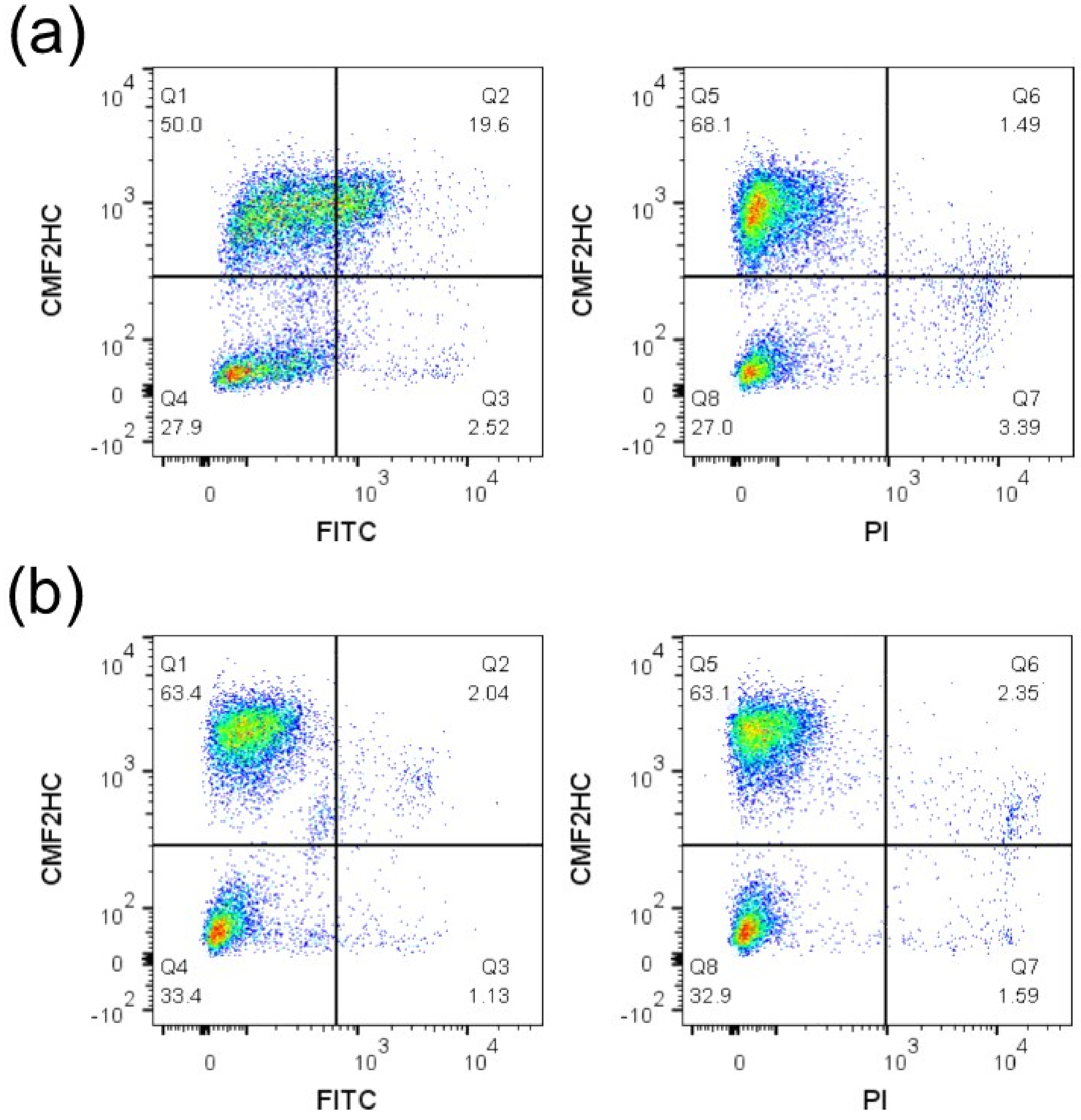

Fig. S21 Flow cytometric analysis of co-culture model of HCT116 and NCM460 cells stained with CellEvent Caspase-3/7 Green Ready Detection Reagent (2.5 μM) after incubation with (a) **1** (2 μM) and (b) solvent control for 24 h. The HCT116 cells were pre-treated with blue-emissive CMF_2HC dye for differentiation from NCM460 cells.

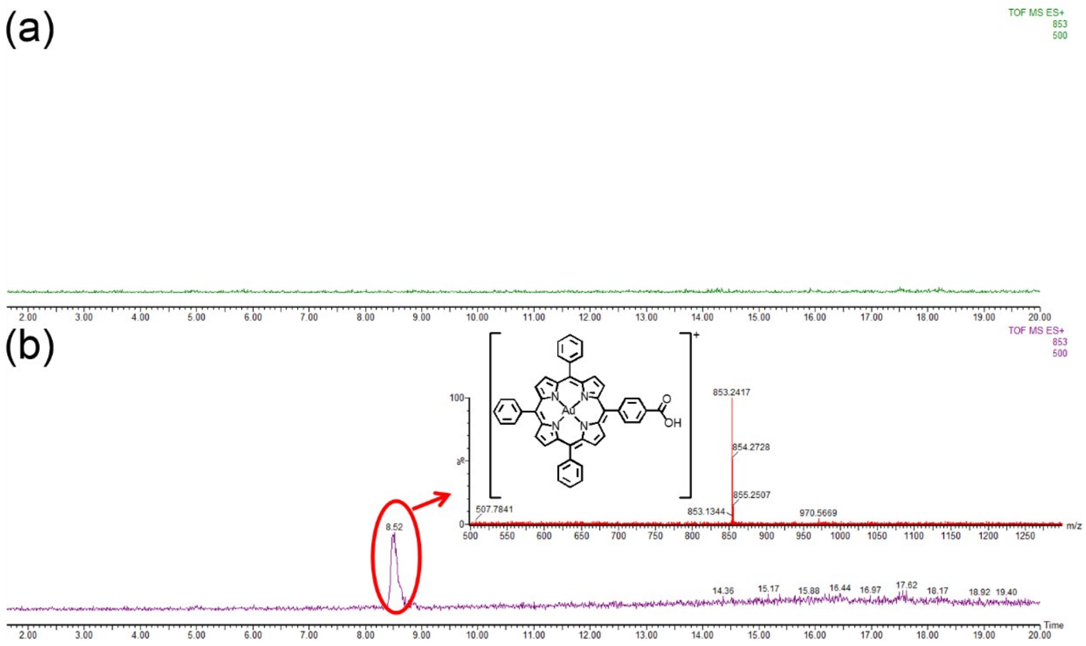

Fig. S22 Selected-ion chromatograms from UPLC-QTOF-MS at *m/z* = 853, corresponding to the *m/z* of $[Au(TPP–COOH)]^{+}$, of homogenized tumor tissues of (a) untreated mice and (b) mice treated by **1** (27 mg/kg), respectively.

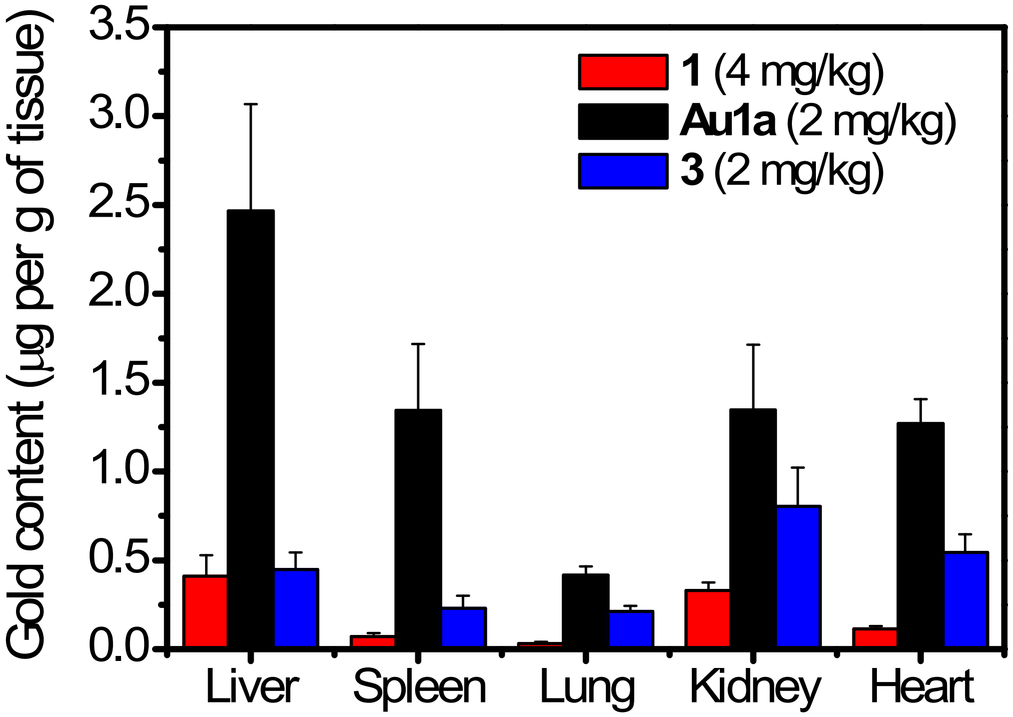

Fig. S23 Biodistribution of gold complexes in nude mice bearing HCT116 xenografts after 14 days of treatment with **1** (4 mg/kg), **Au1a** and **3** (2 mg/kg), respectively, through intravenous injection. The gold content in homogenized tissues was quantified by ICP-MS. *n* = 5 (for **Au1a** and **3**) and 6 (for **1**). Data are shown as mean ± SD.

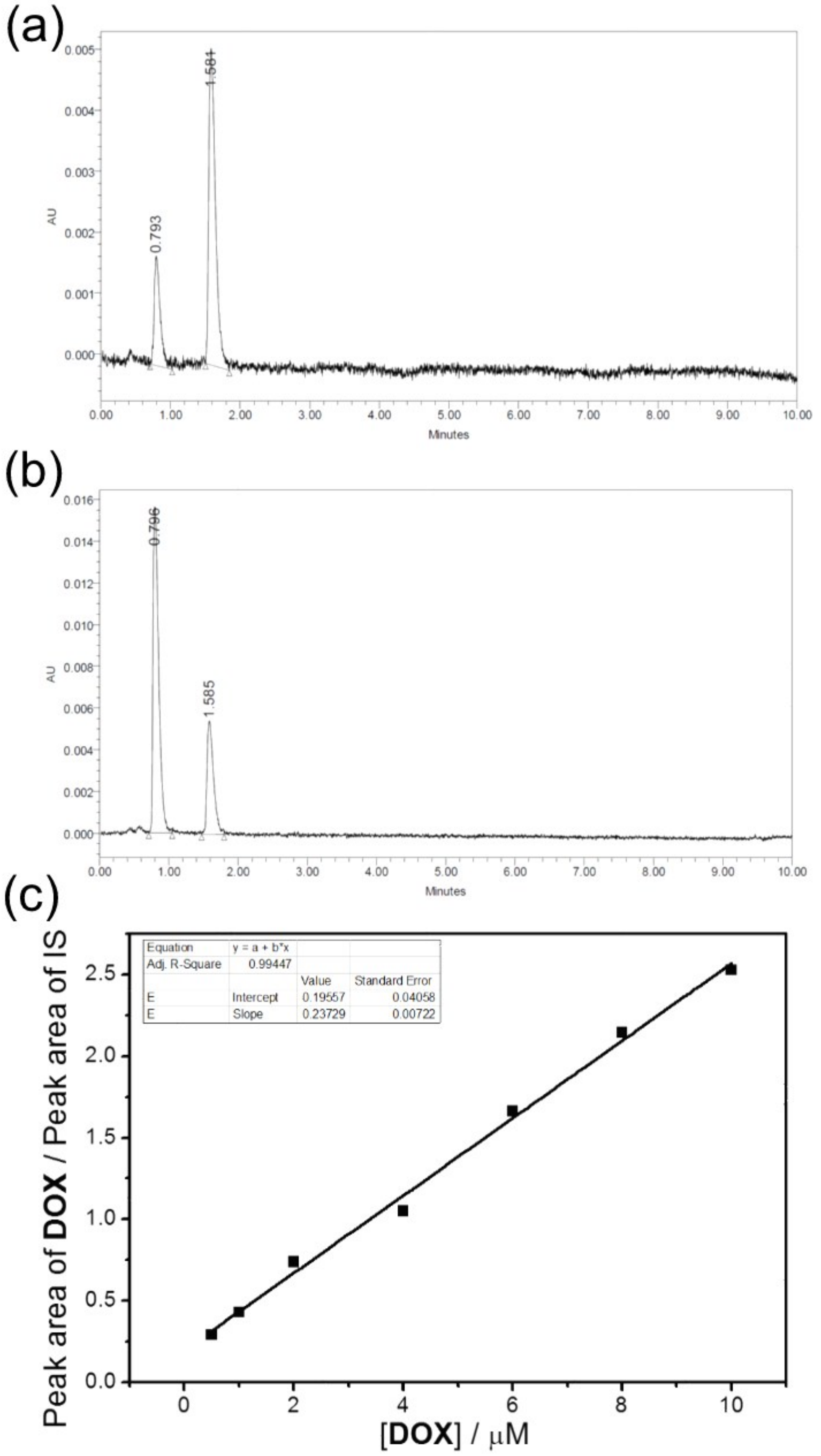

Fig. S24 UPLC traces of (a) nanocomposite of **1** and **DOX** (**NC3**), and (b) **DOX** standard (10 μM), respectively, with daunorubicin hydrochloride (3.6 μM) as internal standard (IS), monitored by UV-vis absorption at 480 nm. (c) Calibration curve of **DOX**.

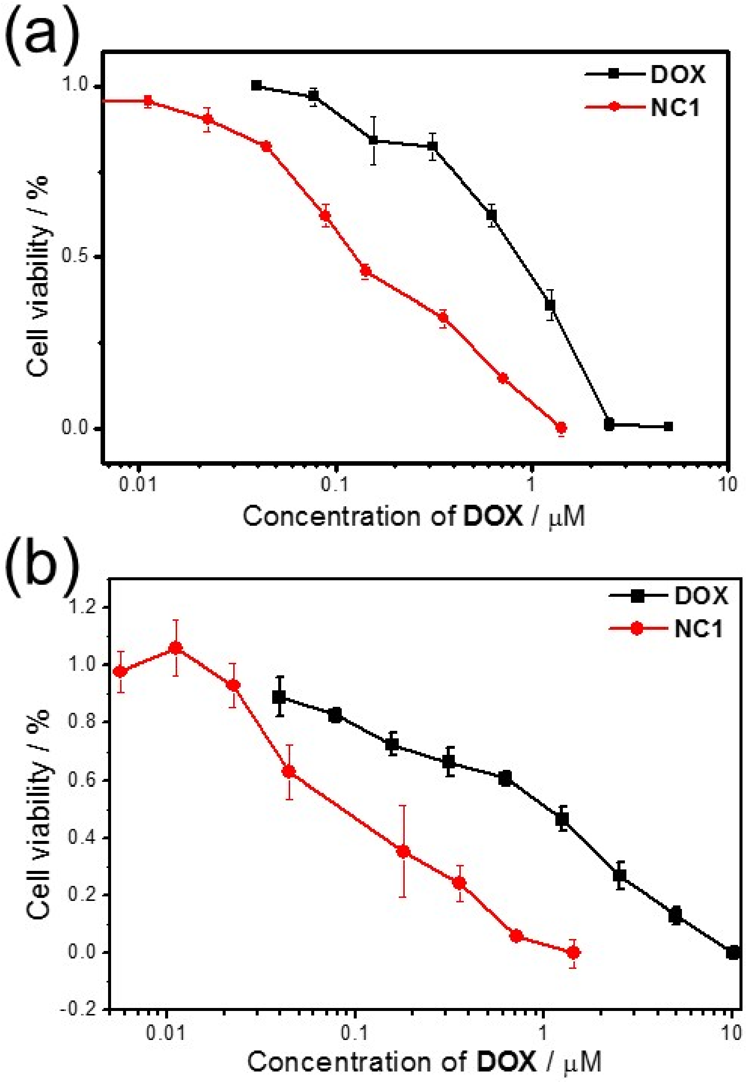

Fig. S25 Profiles showing cell viability of (a) HeLa and (b) A2780adr cells upon treatment with different concentrations of **NC1** and **DOX** for 72 h. **NC1** was composed of **1** and **DOX** ([**1**]:[**DOX**] = 7:1) which were both anti-cancer active.

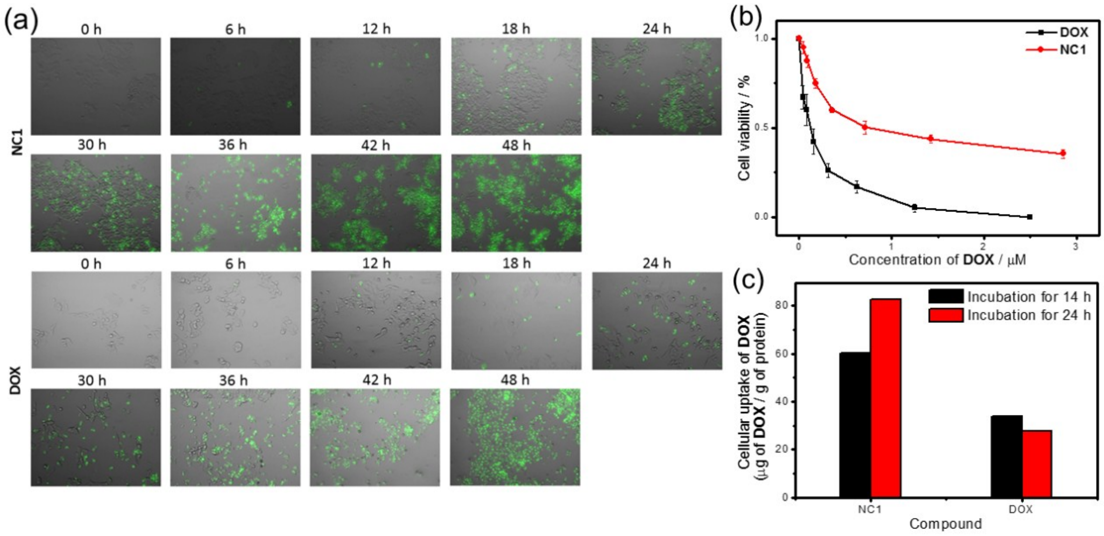

Fig. S26 (a) Fluorescence microscopy images of HCT116 cells after incubation with CellEvent Caspase-3/7 Green ReadyProbes Reagent (30 μL), and **NC1** ([**1**] = 3.5 μM and [**DOX**] = 0.5 μM) and **DOX** (0.5 μM) respectively, at different time intervals. (b) Profile showing cell viability of MIHA cells upon treatment with different concentrations of **NC1** ([**1**]:[**DOX**] = 7:1) and **DOX** for 72 h. (c) Cellular uptake of **NC1** ([**1**] = 3.5 μM and [**DOX**] = 0.5 μM) and **DOX** (0.5 μM) into A2780adr cells, respectively, after incubation at 37 ^{o}C for indicated time intervals.

Table S1 Difference in *in vitro* cytotoxicity toward A2780 and the drug-resistant A2780cis and A2780adr

Compounds	Resistance found in A2780cis[a]	Resistance found in A2780adr[b]
**1**	1.27	2.94
**2**	3.57	> 9.09
**3**	1.0	3.23
**Au1a**	1.49	9.09
**DOX**	1.20	14.3
Cisplatin	3.23	5

[a] Determined by IC_{50} of A2780cis cells / IC_{50} of A2780 cells. [b] Determined by IC_{50} of A2780adr cells / IC_{50} of A2780 cells. Larger values mean stronger resistance found in the drug-resistant A2780cis and A2780adr cells.

Table S2 Relative toxicity of **1–3** and **Au1a** toward cancer cells and NCM460 cells

Compounds/nanostructures	Toxicity toward cancer cells compared to that toward NCM460 cells[a]					
	HeLa	NCI-H460	HCT-116	A2780	A2780cis	A2780adr
**1**	8.6	19.3	42.1	32.2	25.6	10.9
**2**	1.5	2.9	8.6	5.3	1.5	< 0.6
**3**	0.2	1.3	1.4	0.7	0.7	0.2
**Au1a**	0.1	0.3	0.8	0.6	0.4	0.06

[a] Determined by IC_{50} of non-tumorigenic NCM460 cells / IC_{50} of cancer cells. Larger values mean more effective killing of the cancer cells compared to NCM460 cells.

Table S3 Relative toxicity of **1–3** and **Au1a** toward cancer cells and MIHA cells

Compounds/nanostructures	Toxicity toward cancer cells compared to that toward MIHA cells[a]					
	HeLa	NCI-H460	HCT-116	A2780	A2780cis	A2780adr
**1**	7.6	17.1	37.2	28.4	22.7	9.1
**2**	2.5	4.7	14.0	8.6	2.4	< 1.0
**3**	0.9	7.0	7.7	3.5	3.5	1.1
**Au1a**	0.6	1.7	4.4	3.3	3.5	0.4

[a] Determined by IC_{50} of non-tumorigenic MIHA cells / IC_{50} of cancer cells. Larger values mean more effective killing of the cancer cells compared to MIHA cells.

Table S4 Relative toxicity of **1–3** and **Au1a** toward cancer cells and CCD-19Lu cells

Compounds/nanostructures	Toxicity toward cancer cells compared to that toward CCD-19Lu cells[a]					
	HeLa	NCI-H460	HCT-116	A2780	A2780cis	A2780adr
**1**	8.0	17.9	39.0	29.8	23.8	10.1
**3**	0.9	6.6	8.1	3.2	3.8	1.1
**Au1a**	0.3	1.0	2.6	1.9	1.3	0.2

[a] Determined by IC_{50} of non-tumorigenic CCD-19Lu cells / IC_{50} of cancer cells. Larger values mean more effective killing of the cancer cells compared to CCD-19Lu cells.

Table S5 *In vitro* cytotoxicity of **1**, **3** and cisplatin toward non-tumorigenic liver L02 and gliocyte CHEM-5 cells

Compound	IC_{50}[a] / μM	
	L02	CHEM-5
**1**	3.8±0.1	11.3±0.05
**3**	0.2±0.05	3.5±0.03
Cisplatin	5.9±0.02	13.0±0.2

[a] *In vitro* cytotoxicity was determined by MTT assay upon incubation of the live cells with the compounds for 72 h.

Table S6 Relative toxicity of **DOX** toward cancer cells and MIHA cells through administration of **DOX** alone and administration by **NC1**

Compounds/nanostructures	Toxicity toward cancer cells compared to that toward MIHA cells[a]					
	HeLa	NCI-H460	HCT-116	A2780	A2780cis	A2780adr
**DOX** in **NC1**	7.6	17.1	37.2	28.4	22.7	9.1
**DOX** alone	0.2	2.8	0.7	2.2	1.9	0.1

[a] Determined by IC_{50} of non-tumorigenic MIHA cells / IC_{50} of cancer cells. The cytotoxicity of complex **1** in **NC1** has not been taken into considerations. Larger values mean more effective killing of the cancer cells compared to MIHA cells.

Table S7 Relative toxicity of **DOX** toward cancer cells and NCM460 cells through administration of **DOX** alone and administration by **NC1**

Compounds/nanostructures	Toxicity toward cancer cells compared to that toward NCM460 cells[a]					
	HeLa	NCI-H460	HCT-116	A2780	A2780cis	A2780adr
**DOX** in **NC1**	6.4	11.0	50.0	22.5	9.6	8.5
**DOX** alone	1.8	27.8	7.1	22.2	18.5	1.5

[a] Determined by IC_{50} of non-tumorigenic NCM460 cells / IC_{50} of cancer cells. The cytotoxicity of complex **1** in **NC1** has not been taken into considerations. Larger values mean more effective killing of the cancer cells compared to NCM460 cells.
